# Supplementary material for: A Molecular-Wide and Electron Density-Based Approach in Exploring Chemical Reactivity and Explicit Dimethyl Sulfoxide (DMSO) Solvent Molecule Effects in the Proline Catalyzed Aldol Reaction
Source: Molecules. 2022 Jan 31;27(3):962. doi: 10.3390/molecules27030962 (PMC8839911; doi:10.3390/molecules27030962)
Supplement: Supplementary file 1 [file molecules-27-00962-s001.zip › molecules-1548213-supplementary.pdf]

A Molecular-Wide and Electron Density-Based Approach in Exploring Chemical Reactivity and Explicit Dimethyl Sulfoxide (DMSO) Solvent Molecule Effects in the Proline Catalyzed Aldol Reaction

Ignacy Cukrowski*[a], George Dhimba[a] and Darren L. Riley[a]

[a] Department of Chemistry, Faculty of Natural and Agricultural Sciences, University of Pretoria, Lynnwood Road, Pretoria 0002 (South Africa)

[*] Prof Ignacy Cukrowski

E-mail of corresponding author: ignacy.cukrowski@up.ac.za

Dr. George Dhimba, email: u16402902@tuks.co.za

Prof Darren L. Riley, email: darren.riley@up.ac.za

Supplementary Materials

Contents **Page**

Part 1<br>Coordinates and energies of all structures discussed in the main body	S3
Part 2<br>Data pertaining to the 1st and 2nd proton transfers	43
Part 3<br>Search for global minimum and best pre-organized structures of 3-MCs	S48
Part 4<br>Largest net atomic charges in 3-MCs	S54
Part 5<br>Most significant intermolecular diatomic interactions in 3-MCs	S56
Part 6<br>Inter-fragment and selected atom-fragment interaction energies obtained for $\mathcal{A}$, $\mathcal{P}$n and $\mathcal{D}$ in 3-MCs	S72

Part 1

Coordinates and energies of all structures discussed in the main body

1a – the lowest energy conformer (LEC) of proline

Atom	X	Y	Z
C1	-0.0945820881	-1.3616508300	-0.8365335880
C2	-0.5190690347	-1.2573374716	0.6302290991
C3	-1.0788227389	0.1706950832	0.7151583197
C4	-0.1246813852	0.9722544295	-0.1992426764
H5	0.3281244270	0.2480799094	-2.1025552561
H6	0.6591109463	-2.1308210435	-1.0132112033
H7	-0.9632543973	-1.5761338223	-1.4696052990
H8	-1.2490175462	-2.0185574329	0.9075498900
H9	0.3511233168	-1.3671245973	1.2850414934
H10	-2.0880936170	0.2049830909	0.2983008665
H11	-1.1155132690	0.5735847011	1.7271909176
H12	-0.6469204573	1.7777075250	-0.7198264624
N13	0.4628012476	-0.0166263775	-1.1345153508
C14	1.0098594764	1.6334091005	0.6007483932
O15	0.8307496198	2.4944067393	1.4355402361
O16	2.2188399062	1.1665470996	0.2925674012
H17	2.0193456037	0.4865838968	-0.4168367709

Zero-point correction = 0.144439 (Hartree/Particle)
Thermal correction to Energy = 0.151574
Thermal correction to Enthalpy = 0.152518
Thermal correction to Gibbs Free Energy = 0.112465
Sum of electronic and zero-point Energies = –401.167135
Sum of electronic and thermal Energies = –401.160000
Sum of electronic and thermal Enthalpies = –401.159056
Sum of electronic and thermal Free Energies = –401.199110

2 - Acetone

Atom	X	Y	Z
C1	-0.00462863	0.178140532	0.6151523
O2	-0.0134421	0.517341082	1.7864747
C3	-1.28134278	-0.124580786	-0.1338366
H4	-2.13578709	-0.108943756	0.5416987
H5	-1.42267733	0.624186195	-0.9201481
H6	-1.21074387	-1.096426665	-0.6304427
C7	1.28356795	0.038941613	-0.1618913
H8	1.43279417	-1.013549159	-0.4243945
H9	1.22377847	0.594769111	-1.1018739
C10	2.12848123	0.39012183	0.4292614

Zero-point correction =	0.083228 (Hartree/Particle)
Thermal correction to Energy =	0.088510
Thermal correction to Enthalpy =	0.089454
Thermal correction to Gibbs Free Energy =	0.056063
Sum of electronic and zero-point Energies =	–193.146706
Sum of electronic and thermal Energies =	–193.141424
Sum of electronic and thermal Enthalpies=	–193.140480
Sum of electronic and thermal Free Energies=	–193.173871

1b – The higher energy conformer (HEC) of proline

**Atom**	**X**	**Y**	**Z**
C1	-0.4090342829	-1.2417777602	-1.0365123470
C2	-0.6679877535	-1.5447881579	0.4695639391
C3	-0.3162309883	-0.2234293381	1.2092014177
C4	-0.0898466893	0.7677367134	0.0537820277
H5	1.3839410276	-0.3040841776	-0.8583830776
H6	0.0865113893	-2.0562345172	-1.5651403464
H7	-1.3492538419	-1.0266239834	-1.5514139257
H8	-1.7054929740	-1.8402176315	0.6362186313
H9	-0.0336802727	-2.3620827660	0.8174100463
H10	-1.1028450646	0.1090238381	1.8868095774
H11	0.6032172475	-0.3273668019	1.7897031322
H12	-1.0601281261	1.1753315470	-0.2628674836
N13	0.4290475018	-0.0221423670	-1.0821181369
C14	0.7733566709	1.9810686690	0.3373918600
O15	0.9007085432	2.5009165302	1.4196362013
O16	1.3832903879	2.4938242140	-0.7505225570
H17	1.1744272251	1.9208459692	-1.5127589589

Zero-point correction =	0.144477 (Hartree/Particle)
Thermal correction to Energy =	0.151947
Thermal correction to Enthalpy =	0.152891
Thermal correction to Gibbs Free Energy =	0.111845
Sum of electronic and zero-point Energies =	–401.156469
Sum of electronic and thermal Energies =	–401.149000
Sum of electronic and thermal Enthalpies =	–401.148056
Sum of electronic and thermal Free Energies =	–401.189101

3 – The DMSO solvent molecule

Atom	X	Y	Z
C1	0.1390831286	1.3738971442	0.1548933110
H2	-0.3718709417	1.4568834113	1.1151183087
H3	1.1968515605	1.1433203786	0.2883211020
H4	0.0148603640	2.2937622891	-0.4153420520
C5	-0.1941417441	-1.3453122695	0.2887444938
H6	-0.6986171498	-1.2094582639	1.2463671140
H7	-0.5430439196	-2.2588948032	-0.1912406887
H8	0.8895271679	-1.3645337763	0.4117684699
O9	0.2089180421	-0.1281355626	-2.0829901667
S10	-0.6415665170	0.0384712824	-0.8156401979

Zero-point correction=	0.078866 (Hartree/Particle)
Thermal correction to Energy=	0.084550
Thermal correction to Enthalpy=	0.085494
Thermal correction to Gibbs Free Energy=	0.050476
Sum of electronic and zero-point Energies=	–553.208027
Sum of electronic and thermal Energies=	–553.202343
Sum of electronic and thermal Enthalpies=	–553.201399
Sum of electronic and thermal Free Energies=	–553.236418

4a_GMS

Atom	X	Y	Z
C1	-0.6916850048	-1.9375015508	-1.2185793238
C2	-1.6069619830	-2.3973912377	-0.0787230580
C3	-1.6756783939	-1.1474146077	0.8128553526
C4	-1.6270080967	0.0247043311	-0.2081486431
H5	-1.7704851814	-0.4732417091	-2.2069861297
H6	0.3512071852	-1.9667144146	-0.8932061339
H7	-0.7883639362	-2.5209893400	-2.1346306448
H8	-2.5984753746	-2.6468021265	-0.4697230819
H9	-1.2178388612	-3.2685219087	0.4522526545
H10	-2.5713304507	-1.1019025535	1.4316952680
H11	-0.8035236500	-1.1094406505	1.4685078165
H12	-2.6229400284	0.4422444921	-0.3716703728
N13	-1.0753773719	-0.5240601710	-1.4719324254
C14	-0.7413908516	1.1752989962	0.2726118365
O15	-0.9240760916	1.7908845175	1.3008615327
O16	0.2768470597	1.4393638881	-0.5494302064
H17	0.1495733464	0.7766572727	-1.2886132891
C18	2.3804416706	-0.3210758444	1.0129221934
O19	1.7233567346	-1.3294925621	1.2145379515
C20	3.1570755963	-0.1243680716	-0.2663318614
H21	2.8825172843	-0.8796087298	-1.0021978211
H22	2.9830106501	0.8770626844	-0.6662276427
H23	4.2268303159	-0.2050568301	-0.0454639043
C24	2.4480348022	0.7957037052	2.0249368385
H25	3.4689521540	1.1722585878	2.1262586158
H26	1.8279292207	1.6204998632	1.6594133813
H27	2.0658592563	0.4631039696	2.9895110972

Zero-point correction =	0.228970 (Hartree/Particle)
Thermal correction to Energy =	0.243408
Thermal correction to Enthalpy =	0.244352
Thermal correction to Gibbs Free Energy =	0.184586
Sum of electronic and zero-point Energies =	–594.318801
Sum of electronic and thermal Energies =	–594.304363
Sum of electronic and thermal Enthalpies =	–594.303419
Sum of electronic and thermal Free Energies =	–594.363185

4a_p-org

Atom	X	Y	Z
C1	-0.2142578068	-1.3418801271	-0.6664473997
C2	-1.5993364941	-1.9535653225	-0.9065553140
C3	-2.4317407846	-1.3178920232	0.2147961561
C4	-1.8537336948	0.1227623131	0.3059517752
H5	-0.3465398083	0.7035998494	-0.9764160675
H6	0.3093019067	-1.8825469812	0.1290069959
H7	0.4270260249	-1.3405016675	-1.5488502351
H8	-1.9812426733	-1.6456154512	-1.8848729234
H9	-1.5982367073	-3.0445856784	-0.8650357576
H10	-3.5037840457	-1.3096521626	0.0208739079
H11	-2.2644169649	-1.8526202978	1.1544787088
H12	-2.4658115594	0.8145605046	-0.2778743371
N13	-0.4747751384	0.0473074059	-0.2171946258
C14	-1.8626370569	0.6309580569	1.7456262804
O15	-2.8816372374	0.8780779951	2.3574583669
O16	-0.6586507062	0.7624525769	2.3025529247
H17	0.0073932101	0.5297890487	1.6066048547
C18	2.7366003409	-0.0651676578	0.6869122870
O19	2.1165168599	-0.1564435759	1.7337352907
C20	3.5475739128	-1.2165586342	0.1448892824
H21	3.5698117180	-2.0404322372	0.8570093945
H22	3.1006535318	-1.5566501065	-0.7950054992
H23	4.5651123049	-0.8903302141	-0.0880145211
C24	2.7316702357	1.2037349710	-0.1295742178
H25	3.7288412825	1.6549083488	-0.0870051502
H26	2.5269593588	0.9845679163	-1.1807225829
H27	1.9962399911	1.9063231507	0.2584724064

Zero-point correction = 0.228542 (Hartree/Particle)
Thermal correction to Energy = 0.243192
Thermal correction to Enthalpy = 0.244136
Thermal correction to Gibbs Free Energy = 0.183038
Sum of electronic and zero-point Energies = –594.318198
Sum of electronic and thermal Energies = –594.303549
Sum of electronic and thermal Enthalpies = –594.302604
Sum of electronic and thermal Free Energies = –594.363702

5a_TS

Atom	X	Y	Z
C1	-0.0171571315	-1.9617347208	-0.4179515031
C2	-1.5104260512	-2.1161066394	-0.6847728431
C3	-2.1145567745	-1.0140397845	0.1894788547
C4	-1.0800479450	0.1488501252	0.1230439600
H5	0.3431549380	-0.1531409416	-1.3248784554
H6	0.2598532175	-2.3563482371	0.5630904862
H7	0.6209771353	-2.4107147098	-1.1757246461
H8	-1.7246885314	-1.9372197639	-1.7423963487
H9	-1.8774877917	-3.1095480543	-0.4237611380
H10	-3.0968917020	-0.6779127909	-0.1387118297
H11	-2.2054524682	-1.3678063925	1.2198196968
H12	-1.3776079701	0.8826085825	-0.6228925990
N13	0.2084380865	-0.4874363814	-0.3744340564
C14	-1.1222967116	0.8851515543	1.4752560128
O15	-2.0335630020	1.6895119170	1.6199679096
O16	-0.3030363747	0.5738758265	2.4421832989
H17	0.6192000107	0.0398139417	2.1519041614
C18	1.8651856094	-0.1017953435	0.4842933168
O19	1.7094576568	-0.5393201226	1.6910288907
C20	2.8380475301	-0.8893770490	-0.3759160618
H21	2.7271615466	-1.9591519660	-0.2043707467
H22	2.7394021364	-0.6660248145	-1.4397873798
H23	3.8453066734	-0.5990076149	-0.0632659391
C24	1.9416025032	1.4052948871	0.2627397574
H25	2.9179841381	1.7318104600	0.6322268536
H26	1.8744880872	1.6693386528	-0.7941016133
H27	1.1798531848	1.9452293796	0.8226319615

Zero-point correction =	0.229618 (Hartree/Particle)
Thermal correction to Energy =	0.241264
Thermal correction to Enthalpy =	0.242208
Thermal correction to Gibbs Free Energy =	0.192049
Sum of electronic and zero-point Energies =	–594.300687
Sum of electronic and thermal Energies =	–594.289042
Sum of electronic and thermal Enthalpies =	–594.288098
Sum of electronic and thermal Free Energies =	–594.338256

5a_eq

Atom	X	Y	Z
C1	-0.1603524393	-1.5611266845	0.9399083284
C2	-1.6587996187	-1.7059470406	0.6222442348
C3	-1.7869742887	-1.2988729275	-0.8548002985
C4	-0.7551554935	-0.1738990383	-1.0005381376
H5	0.7958921265	-1.4796061116	-0.8835772537
H6	0.0371684123	-0.9891542427	1.8409757273
H7	0.3593910856	-2.5145947462	0.9964298973
H8	-2.0088865609	-2.7212415294	0.8055698201
H9	-2.2398111414	-1.0291770444	1.2509376054
H10	-1.5259016630	-2.1327896799	-1.5128308850
H11	-2.7813104438	-0.9496536568	-1.1249600856
H12	-0.4340085535	-0.0265851489	-2.0295651408
N13	0.4305112578	-0.7822587489	-0.2340376859
C14	-1.3269921850	1.1783591290	-0.4810370308
O15	-2.2443068597	1.6205342628	-1.1914411544
O16	-0.8618964088	1.6943083531	0.5751585493
H17	0.5754507787	1.3025383786	1.1139162385
C18	1.7006089781	0.0987347134	0.1254193602
O19	1.4371168523	0.8172042996	1.2746771558
C20	1.9993966296	0.9840846083	-1.0835998143
H21	1.2171637880	1.7273717131	-1.2355518816
H22	2.9334213687	1.5112627790	-0.8852576306
H23	2.1232021099	0.3965009921	-1.9960270247
C24	2.8361320230	-0.8757807549	0.4182230516
H25	3.0706191044	-1.4981672330	-0.4477161235
H26	3.7220174294	-0.2921002793	0.6704665720
H27	2.5930037120	-1.5108443624	1.2704136064

Zero-point correction =	0.234574 (Hartree/Particle)
Thermal correction to Energy =	0.246325
Thermal correction to Enthalpy =	0.247270
Thermal correction to Gibbs Free Energy =	0.197264
Sum of electronic and zero-point Energies =	–594.309671
Sum of electronic and thermal Energies =	–594.297919
Sum of electronic and thermal Enthalpies =	–594.296975
Sum of electronic and thermal Free Energies =	–594.346980

5a_p-org

Atom	X	Y	Z
C1	0.4722714307	-1.7931193190	-0.9699678577
C2	-0.4084548344	-2.3899837540	0.1522703504
C3	-1.3985562225	-1.2621027291	0.5513734610
C4	-1.2529491073	-0.2406661687	-0.5694321616
H5	0.3577413833	0.1187470566	-1.8156092944
H6	1.5325036959	-2.0135683130	-0.8949277460
H7	0.1315259609	-2.1063362616	-1.9558229237
H8	-0.9349600091	-3.2640493246	-0.2303796979
H9	0.1938721403	-2.7147355704	0.9995090589
H10	-2.4267311843	-1.6108980450	0.6183435781
H11	-1.1386506982	-0.8106335601	1.5099483000
H12	-1.7114743236	-0.6581651505	-1.4734605802
N13	0.2396286985	-0.2982596936	-0.8925697359
C14	-1.8843429405	1.1669184142	-0.4665066339
O15	-2.9801121548	1.2193441845	0.1120313904
O16	-1.2627192158	2.1007540316	-1.0592171982
H17	0.1199172494	2.0588027397	-0.0518459130
C18	1.2892098462	0.5044542251	0.0353783839
O19	0.6424706199	1.5721016590	0.6303783040
C20	2.3781341306	0.9466297365	-0.9418450452
H21	1.9705523324	1.6400320807	-1.6823297266
H22	3.1557102360	1.4642788739	-0.3796567208
H23	2.8303524838	0.0966710353	-1.4575571574
C24	1.8405738948	-0.3529445445	1.1673319674
H25	2.4034459947	-1.2162659385	0.8185750861
H26	2.5241135899	0.2856382542	1.7283907884
H27	1.0507270033	-0.6705439186	1.8445977239

Zero-point correction =	0.234647 (Hartree/Particle)
Thermal correction to Energy =	0.246415
Thermal correction to Enthalpy =	0.247359
Thermal correction to Gibbs Free Energy =	0.197330
Sum of electronic and zero-point Energies =	–594.296720
Sum of electronic and thermal Energies =	–594.284952
Sum of electronic and thermal Enthalpies =	–594.284008
Sum of electronic and thermal Free Energies =	–594.334037

6a_TS

Atom	X	Y	Z
C1	0.3840649949	-1.6807530894	-1.1254104814
C2	-0.4296596300	-2.4017196034	0.0076021589
C3	-1.3647230266	-1.3097792510	0.6350233223
C4	-1.2628190561	-0.2419604123	-0.4347763148
H5	-0.1615703227	0.7968619808	-1.5892177371
H6	1.4410339258	-1.9318985223	-1.1383940911
H7	-0.0245704768	-1.9060248712	-2.1105153911
H8	-1.0179709031	-3.2118135273	-0.4248388703
H9	0.2296125906	-2.8396732140	0.7562331855
H10	-2.3865417351	-1.6666041934	0.7588184012
H11	-1.0074045981	-0.9576618061	1.6019462897
H12	-1.7738342961	-0.6641265278	-1.3130267784
N13	0.1733214716	-0.2243149459	-0.8690517737
C14	-1.7122547391	1.2167690212	-0.5345692081
O15	-2.6427349748	1.7256654320	0.0663782109
O16	-0.9778565964	1.7694267751	-1.4763114182
H17	0.5018658321	2.2154649446	0.2179058519
C18	1.2362249533	0.4017566503	0.0863071479
O19	0.6469257400	1.4632234373	0.8069227220
C20	2.3456705639	0.9025168979	-0.8406478492
H21	1.9586718254	1.6555060344	-1.5319281176
H22	3.1430460117	1.3489083176	-0.2449282725
H23	2.7652465233	0.0832161630	-1.4273067950
C24	1.7991649574	-0.5359313787	1.1545645389
H25	2.2807273640	-1.4141821813	0.7291181010
H26	2.5551333010	0.0263715307	1.7041954333
H27	1.0349302998	-0.8467436608	1.8628077349

Zero-point correction =	0.229737 (Hartree/Particle)
Thermal correction to Energy =	0.241029
Thermal correction to Enthalpy =	0.241973
Thermal correction to Gibbs Free Energy =	0.193718
Sum of electronic and zero-point Energies =	–594.270035
Sum of electronic and thermal Energies =	–594.258743
Sum of electronic and thermal Enthalpies =	–594.257799
Sum of electronic and thermal Free Energies =	–594.306054

6a_eq

Atom	X	Y	Z
C1	0.5114657874	-1.6413051547	-1.2748653443
C2	-0.2645314974	-2.4937815007	-0.2316325121
C3	-1.3103889259	-1.5206633124	0.3650321054
C4	-1.1237636008	-0.2422774071	-0.4783541488
H5	-1.5991368613	1.8429498781	-1.5802733636
H6	1.5711868715	-1.8772173809	-1.3168667209
H7	0.0987974020	-1.8179231343	-2.2727226806
H8	-0.7322100510	-3.3558393303	-0.7105899673
H9	0.4020632976	-2.8752267807	0.5435264316
H10	-2.3328322826	-1.8928056827	0.2802955050
H11	-1.1216331218	-1.3157208973	1.4176800683
H12	-1.7169223291	-0.3713051422	-1.3933000808
N13	0.2872060490	-0.2139892093	-0.9461491176
C14	-1.6515939214	1.0394671019	0.1622730874
O15	-1.9376436977	1.1562182589	1.3282816661
O16	-1.9108389746	2.0572860657	-0.6898630358
H17	0.9690079951	2.1961151620	-0.8810532573
C18	1.3049899153	0.4423071152	-0.0816281577
O19	0.9351360334	1.8232834294	0.0092655666
C20	2.6802562945	0.3389612605	-0.7536415676
H21	2.6082448845	0.6028825337	-1.8116348515
H22	3.3552586729	1.0383176514	-0.2578501897
H23	3.1119764304	-0.6589992474	-0.6695668673
C24	1.3794211188	-0.0379761750	1.3726442635
H25	1.5838010310	-1.1081096210	1.4131826178
H26	2.1879560050	0.4858697471	1.8866438888
H27	0.4481274751	0.1714817722	1.897566662

Zero-point correction =	0.232583 (Hartree/Particle)
Thermal correction to Energy =	0.244807
Thermal correction to Enthalpy =	0.245751
Thermal correction to Gibbs Free Energy =	0.195193
Sum of electronic and zero-point Energies =	–594.290553
Sum of electronic and thermal Energies =	–594.278329
Sum of electronic and thermal Enthalpies =	–594.277385
Sum of electronic and thermal Free Energies =	–594.327943

4b_GMS

Atom	X	Y	Z
C1	-2.2641727131	0.5704324091	-2.2935412506
C2	-2.0183714657	1.5842351092	-1.1681108589
C3	-0.8059885576	0.9801408678	-0.4439682103
C4	-0.9740779599	-0.5687808272	-0.6636731390
H5	-2.8582022975	-1.0642012062	-1.2137865744
H6	-3.2712514758	0.6079634282	-2.7124366242
H7	-1.5537592881	0.7355269124	-3.1115116773
H8	-1.8282619419	2.5974992927	-1.5286952714
H9	-2.8862263879	1.6138216175	-0.5022746603
H10	0.1252672965	1.3075405241	-0.9106044184
H11	-0.7610090224	1.2544835081	0.6109148953
H12	-0.0230654376	-0.9870289409	-1.0036226683
N13	-2.0144019294	-0.7549819190	-1.6896299842
C14	-1.3453822750	-1.2676302056	0.6389173393
O15	-2.4684636724	-1.6472373097	0.9006609884
O16	-0.3565017639	-1.4231858963	1.5223523830
H17	0.5146478782	-1.0923819410	1.1767835190
C18	2.5928144812	0.4425407195	0.8724470209
O19	1.9985879889	-0.5861617150	0.5635727742
C20	2.0666762043	1.3731017646	1.9301715900
H21	1.7603282723	2.3101156938	1.4538636000
H22	1.2171640502	0.9375330099	2.4539337205
H23	2.8588761693	1.6234335351	2.6401492003
C24	3.8783079403	0.8204182318	0.1938785126
H25	3.8232290415	1.8504326052	-0.1691391111
H26	4.6912234572	0.7862626391	0.9265267231
H27	4.0954134082	0.1396080928	-0.6274778183

Zero-point correction =	0.228937 (Hartree/Particle)
Thermal correction to Energy =	0.243389
Thermal correction to Enthalpy =	0.244334
Thermal correction to Gibbs Free Energy =	0.183671
Sum of electronic and zero-point Energies =	–594.317076
Sum of electronic and thermal Energies =	–594.302624
Sum of electronic and thermal Enthalpies =	–594.301680
Sum of electronic and thermal Free Energies =	–594.362342

4b_p-org

Atom	X	Y	Z
C1	-0.1705743733	2.2625588835	-0.2296233115
C2	-1.6761737669	2.5380035918	0.0194944711
C3	-2.2901023528	1.1345282590	0.2746682983
C4	-1.0790489148	0.1804647631	0.2294733497
H5	-0.3068646154	0.7383195994	-1.5847054566
H6	0.2691896879	2.8933345220	-1.0034665566
H7	0.3989802266	2.4155604209	0.6925745741
H8	-1.8230059502	3.2141048018	0.8641064264
H9	-2.1328889599	3.0030084112	-0.8568177672
H10	-2.8138662671	1.0674281728	1.2299900201
H11	-2.9954616580	0.8632950354	-0.5118461906
H12	-0.6485011465	0.0793254136	1.2320526387
N13	-0.0488869504	0.8401142533	-0.6048232405
C14	-1.3742978437	-1.2264964519	-0.2913608127
O15	-2.3747288993	-1.5187670048	-0.9110364716
O16	-0.4444313465	-2.1536309177	-0.0417117532
H17	0.3372161545	-1.7845212918	0.4494772794
C18	2.3797330493	-0.3848354803	0.4472006969
O19	1.6399069737	-1.0241979952	1.1921448964
C20	2.6822270222	-0.8422165771	-0.9536016125
H21	1.9475290966	-1.5684235454	-1.2967404908
H22	2.7247829471	0.0044841248	-1.6392909278
H23	3.6721920945	-1.3134559482	-0.9451927161
C24	3.0858460230	0.8480144796	0.9339154646
H25	4.1672709971	0.7269121054	0.8181619936
H26	2.7906698696	1.6961909234	0.3104755157
H27	2.8403909027	1.0490004512	1.9756416826

Zero-point correction = 0.229360 (Hartree/Particle)
Thermal correction to Energy = 0.243496
Thermal correction to Enthalpy = 0.244441
Thermal correction to Gibbs Free Energy = 0.186547
Sum of electronic and zero-point Energies = –594.313547
Sum of electronic and thermal Energies = –594.299411
Sum of electronic and thermal Enthalpies = –594.298466
Sum of electronic and thermal Free Energies = –594.356360

5b_TS

Atom	X	Y	Z
C1	-0.4029099281	1.3024110333	-1.3257489985
C2	-1.9174399546	1.1114922786	-1.2901921809
C3	-2.1834922370	0.8312475511	0.1981415825
C4	-0.9399721225	0.0612790829	0.6793408962
H5	0.1943058859	-0.6154258908	-0.9217070965
H6	0.0561870105	1.1769755716	-2.3047158504
H7	-0.1273985150	2.2768937074	-0.9233666977
H8	-2.4507093678	1.9873852726	-1.6609547142
H9	-2.2025856696	0.2523472733	-1.9039702688
H10	-2.2678017114	1.7688597043	0.7506649607
H11	-3.0878022452	0.2499351600	0.3684762229
H12	-0.5325898950	0.4806547448	1.6016662342
N13	0.1122124629	0.2513814606	-0.3926802541
C14	-1.1493198352	-1.4420429328	0.9512781460
O15	-2.2523012523	-1.9540080953	0.9621994976
O16	-0.0446733842	-2.1218056864	1.1794168958
H17	0.8046998199	-1.4830678168	1.2384240659
C18	1.7889211545	0.3746539540	0.3300891472
O19	1.7806308693	-0.4765103396	1.3163419522
C20	1.9013903880	1.8343898516	0.7458282002
H21	1.9060922502	2.5253085689	-0.0977119907
H22	1.1037110631	2.1040017957	1.4408652989
H23	2.8521357775	1.9371285989	1.2747447019
C24	2.6385386973	0.0005673327	-0.8805560437
H25	2.4516086592	0.6421630568	-1.7440071915
H26	3.6876550476	0.1113665282	-0.5934985386
H27	2.4739070320	-1.0445817657	-1.1523679766

Zero-point correction = 0.230689 (Hartree/Particle)
Thermal correction to Energy = 0.242251
Thermal correction to Enthalpy = 0.243195
Thermal correction to Gibbs Free Energy = 0.193182
Sum of electronic and zero-point Energies = –594.302870
Sum of electronic and thermal Energies = –594.291308
Sum of electronic and thermal Enthalpies = –594.290364
Sum of electronic and thermal Free Energies = –594.340377

5b_eq

Atom	X	Y	Z
C1	-0.3093767288	1.0397269453	-1.4207433194
C2	-1.8304584926	0.9978867458	-1.2953874471
C3	-2.0460736433	1.0078190952	0.2237853641
C4	-0.9464056075	0.0785582145	0.7513967534
H5	0.1604945402	-0.8367186830	-0.6044614963
H6	0.0830306840	0.6544180127	-2.3589974143
H7	0.0677758136	2.0475499194	-1.2583573451
H8	-2.2940884610	1.8436074136	-1.8030670647
H9	-2.2223914799	0.0775699166	-1.7361995974
H10	-1.9090971057	2.0178113930	0.6182649102
H11	-3.0299790730	0.6532052101	0.5271947259
H12	-0.5645824023	0.3675162666	1.7258979511
N13	0.1696130890	0.1627216567	-0.2845289097
C14	-1.3653211282	-1.4238718667	0.8260269206
O15	-2.2686926995	-1.6975288939	1.6340280314
O16	-0.7322979609	-2.1991396262	0.0530376434
H17	1.8432089004	-1.4054178552	0.9529364658
C18	1.5881531700	0.4171985520	0.2664039693
O19	1.7746888382	-0.5094075679	1.3097579027
C20	1.6989050150	1.8018486852	0.8858444600
H21	1.5830808106	2.5890799146	0.1424441601
H22	0.9629832456	1.9436232260	1.6775949174
H23	2.6932337290	1.8914971152	1.3238205334
C24	2.5791611458	0.1974166469	-0.8724772391
H25	2.4522935387	0.9365720409	-1.6640801261
H26	3.5887621219	0.2909511223	-0.4714316655
H27	2.4664801407	-0.8013935996	-1.3030030842

Zero-point correction = 0.234061 (Hartree/Particle)
Thermal correction to Energy = 0.246190
Thermal correction to Enthalpy = 0.247135
Thermal correction to Gibbs Free Energy = 0.195918
Sum of electronic and zero-point Energies = –594.313838
Sum of electronic and thermal Energies = –594.301708
Sum of electronic and thermal Enthalpies = –594.300764
Sum of electronic and thermal Free Energies = –94.3519810

6b_TS

Atom	X	Y	Z
C1	-0.2904715225	0.9838139059	-1.4196713284
C2	-1.8149650931	0.9955604922	-1.2892345895
C3	-2.0271383548	1.0514628972	0.2303058807
C4	-0.9324878909	0.1212379562	0.7653786955
H5	-0.0109712243	-1.1168594758	-0.4546821646
H6	0.0677018400	0.5381292459	-2.3465734295
H7	0.1062718851	1.9979639595	-1.3418893946
H8	-2.2592749475	1.8394684401	-1.8172032231
H9	-2.2394421723	0.0762273606	-1.7022450837
H10	-1.8596541476	2.0657550685	0.6011262606
H11	-3.0192103283	0.7306738217	0.5477791686
H12	-0.5809358965	0.3870550280	1.7591518630
N13	0.1726818060	0.1630038622	-0.2544570093
C14	-1.3561552668	-1.3721986990	0.7848170475
O15	-2.2383952230	-1.7874553696	1.5233606451
O16	-0.6623498087	-2.0625163538	-0.0724158385
H17	1.8882642924	-1.3925524188	0.9393538602
C18	1.5611563603	0.4286486574	0.2696050840
O19	1.7905308224	-0.5077057816	1.3141350033
C20	1.6930762776	1.8076477177	0.9088348054
H21	1.5026324334	2.6012281807	0.1865603497
H22	1.0042721313	1.9185633631	1.7472645407
H23	2.7103859126	1.9237487666	1.2846685743
C24	2.5560984931	0.2267600654	-0.8721019139
H25	2.4343044337	0.9820082839	-1.6496665547
H26	3.5681719695	0.3040784385	-0.4729123532
H27	2.4291032190	-0.7606474124	-1.3238888956

Zero-point correction =	0.229342 (Hartree/Particle)
Thermal correction to Energy =	0.241006
Thermal correction to Enthalpy =	0.241950
Thermal correction to Gibbs Free Energy =	0.192443
Sum of electronic and zero-point Energies =	–594.311156
Sum of electronic and thermal Energies =	–594.299492
Sum of electronic and thermal Enthalpies =	–594.298548
Sum of electronic and thermal Free Energies =	–594.348055

6b_eq

Atom	X	Y	Z
C1	-0.2608052919	1.0213456904	-1.4225500310
C2	-1.7871631051	0.9962275516	-1.3276666580
C3	-2.0242453754	1.0351967108	0.1872570910
C4	-0.8996048405	0.1326060263	0.7337562740
H5	-0.1836369914	-1.5768179484	-0.4857147364
H6	0.1177070659	0.6106558699	-2.3589359143
H7	0.1018105229	2.0518904404	-1.3290859007
H8	-2.2461009247	1.8332597776	-1.8549923090
H9	-2.1817087136	0.0696657724	-1.7555725005
H10	-1.8785725983	2.0500107754	0.5653743982
H11	-3.0143198036	0.6970051912	0.4931125567
H12	-0.5692875304	0.4490818048	1.7225826512
N13	0.1865414414	0.1875833991	-0.2756571013
C14	-1.3705642127	-1.3209043281	0.8906618703
O15	-2.1903171150	-1.6753473824	1.7100464395
O16	-0.8061247925	-2.1664887088	0.0269190724
H17	1.8898329401	-1.4018251731	0.8501400974
C18	1.5520402004	0.4415116402	0.2520323040
O19	1.8022327142	-0.5354952959	1.2670314038
C20	1.6997271603	1.7958614273	0.9513163871
H21	1.4908551140	2.6149754372	0.2620491100
H22	1.0246693457	1.8742545758	1.8051341596
H23	2.7222942507	1.9061662842	1.3157724709
C24	2.5623679489	0.2927459223	-0.8866791455
H25	2.4505037151	1.0833548720	-1.6304138273
H26	3.5715586671	0.3487023032	-0.4757541811
H27	2.4335102083	-0.6720226354	-1.3847639811

Zero-point correction =	0.232629 (Hartree/Particle)
Thermal correction to Energy =	0.244708
Thermal correction to Enthalpy =	0.245652
Thermal correction to Gibbs Free Energy =	0.195050
Sum of electronic and zero-point Energies =	–594.313014
Sum of electronic and thermal Energies =	–594.300935
Sum of electronic and thermal Enthalpies =	–594.299990
Sum of electronic and thermal Free Energies =	–594.350592

Data pertaining to 3-MCs

4A_inp-1

Atom	X	Y	Z
C1	-0.27077	-1.78594	-1.73105
C2	-1.34838	-2.41046	-0.83594
C3	-1.67841	-1.26699	0.13731
C4	-1.50108	0.00752	-0.73527
H5	-1.23821	-0.2698	-2.76472
H6	0.69742	-1.81919	-1.22405
H7	-0.1701	-2.26856	-2.7044
H8	-2.22687	-2.67275	-1.43281
H9	-1.00475	-3.31066	-0.32196
H10	-2.68227	-1.33022	0.5572
H11	-0.96279	-1.25922	0.96166
H12	-2.46979	0.40375	-1.05174
N13	-0.68709	-0.37367	-1.90997
C14	-0.79861	1.14059	0.01603
O15	-1.20633	1.63822	1.04489
O16	0.32965	1.53167	-0.57739
H17	0.36638	0.92566	-1.73105
C18	2.22388	-0.31814	1.15325
O19	1.5818	-1.35439	1.20736
C20	3.17096	-0.0324	0.01293
H21	3.03964	-0.76076	-0.78669
H22	3.01404	0.97998	-0.36614
H23	4.19958	-0.08441	0.38552
C24	2.10221	0.74596	2.21581
H25	3.08369	1.14076	2.49018
H26	1.51963	1.5734	1.79867
	1.58916	0.35464	3.09372
S28	-3.7352	-0.35368	-4.65722
C29	-4.36649	-1.40493	-3.30614
H30	-4.16543	-0.92569	-2.34776
H31	-5.43754	-1.54942	-3.45335
H32	-3.84691	-2.35934	-3.37818
C33	-4.63693	1.16965	-4.21975
H34	-4.33199	1.93466	-4.9327
H35	-5.70749	0.97996	-4.30828
H36	-4.37039	1.4617	-3.20347
O37	-2.25435	-0.07826	-4.33818

Zero-point correction=	0.309247 (Hartree/Particle)
Thermal correction to Energy=	0.331115
Thermal correction to Enthalpy=	0.332059
Thermal correction to Gibbs Free Energy=	0.253265
Sum of electronic and zero-point Energies=	–1147.536703
Sum of electronic and thermal Energies=	–1147.514835
Sum of electronic and thermal Enthalpies=	–1147.513891
Sum of electronic and thermal Free Energies=	–1147.592685

4A_LM-1

Atom	X	Y	Z
C1	1.84745	0.38565	1.60019
C2	2.69765	1.60966	1.25826
C3	1.63510	2.66761	0.92111
C4	0.51912	1.84654	0.22680
H5	-0.09611	-0.03338	0.88515
H6	2.38993	-0.55719	1.53015
H7	1.44483	0.47263	2.61762
H8	3.35116	1.91202	2.07763
H9	3.32190	1.39878	0.38421
H10	1.23759	3.10597	1.83921
H11	2.00144	3.47989	0.29298
H12	-0.47028	2.19376	0.53489
N13	0.76514	0.43455	0.59242
C14	0.56737	1.96828	-1.30389
O15	0.34734	2.99391	-1.91370
O16	0.87661	0.81753	-1.89825
H17	0.98653	0.20828	-1.09584
C18	1.58371	-2.71561	-0.14921
O19	1.62110	-3.01012	1.03374
C20	0.27525	-2.68307	-0.90387
H21	-0.52793	-2.37797	-0.23350
H22	0.31507	-2.02786	-1.77566
H23	0.07106	-3.69763	-1.26472
C24	2.83742	-2.38760	-0.92278
H25	2.86403	-2.94969	-1.86073
H26	2.82558	-1.32603	-1.19176
H27	3.72536	-2.59842	-0.32799
S28	-3.17493	-0.61899	0.50595
C29	-3.58083	1.15234	0.66151
H30	-2.77927	1.74948	0.22666
H31	-4.52599	1.33589	0.14876
H32	-3.68531	1.36077	1.72547
C33	-2.87133	-0.63770	-1.29392
H34	-2.58418	-1.65333	-1.56104
H35	-3.79704	-0.36533	-1.80250
H36	-2.07099	0.06321	-1.53153
O37	-1.80401	-0.81346	1.17967

Zero-point correction=	0.309232 (Hartree/Particle)
Thermal correction to Energy=	0.331000
Thermal correction to Enthalpy=	0.331944
Thermal correction to Gibbs Free Energy=	0.253731
Sum of electronic and zero-point Energies=	–1147.537023
Sum of electronic and thermal Energies=	–1147.515254
Sum of electronic and thermal Enthalpies=	–1147.514310
Sum of electronic and thermal Free Energies=	–1147.592523

4A_LM-2

Atom	X	Y	Z
C1	1.90654	0.80786	-1.67917
C2	3.30525	0.25076	-1.40287
C3	3.01068	-1.20473	-1.01246
C4	1.69347	-1.08705	-0.21081
H5	0.10342	0.01102	-0.95579
H6	1.86046	1.89506	-1.60191
H7	1.57541	0.52284	-2.68597
H8	3.96963	0.33628	-2.26384
H9	3.76427	0.78550	-0.56518
H10	2.83314	-1.80584	-1.90750
H11	3.80488	-1.68105	-0.43730
H12	1.04618	-1.95100	-0.38565
N13	1.06028	0.17584	-0.64029
C14	1.94531	-1.03888	1.30382
O15	2.45141	-1.94696	1.93181
O16	1.54810	0.09411	1.87578
H17	1.16709	0.62431	1.11491
C18	-1.00585	2.69772	0.51744
O19	-0.78751	2.09977	1.55901
C20	0.01778	3.62274	-0.09237
H21	0.99688	3.46645	0.35885
H22	0.06795	3.48850	-1.17534
H23	-0.30152	4.65569	0.08585
C24	-2.30469	2.53858	-0.23017
H25	-2.63064	3.48344	-0.67053
H26	-2.12919	1.82845	-1.04676
H27	-3.07723	2.13386	0.42352
S28	-2.68629	-1.11757	-0.33837
C29	-2.81334	-2.91034	-0.64068
H30	-1.81143	-3.34124	-0.63498
H31	-3.43796	-3.35137	0.13734
H32	-3.28208	-3.03764	-1.61566
C33	-1.82334	-1.19708	1.26606
H34	-1.54124	-0.17667	1.52485
H35	-2.51552	-1.59960	2.00690
H36	-0.94508	-1.83601	1.17272
O37	-1.65667	-0.58878	-1.35683

Zero-point correction=	0.309732 (Hartree/Particle)
Thermal correction to Energy=	0.331197
Thermal correction to Enthalpy=	0.332141
Thermal correction to Gibbs Free Energy=	0.256013
Sum of electronic and zero-point Energies=	–1147.538803
Sum of electronic and thermal Energies=	–1147.517337
Sum of electronic and thermal Enthalpies=	–1147.516393
Sum of electronic and thermal Free Energies=	–1147.592521

4A_LM-3

Atom	X	Y	Z
C1	1.10226	-0.00349	-1.01483
C2	2.46431	-0.70368	-1.03161
C3	2.41761	-1.55980	0.24321
C4	1.66477	-0.64855	1.23970
H5	-0.13475	0.22542	0.65742
H6	1.09310	0.92622	-1.58688
H7	0.32805	-0.66315	-1.42582
H8	2.62135	-1.29328	-1.93583
H9	3.26959	0.03476	-0.96518
H10	1.82854	-2.46365	0.06898
H11	3.39802	-1.86088	0.61290
H12	1.05182	-1.23000	1.93324
N13	0.85780	0.27436	0.41953
C14	2.63596	0.17239	2.10437
O15	3.40865	-0.31602	2.90357
O16	2.55350	1.48299	1.88913
H17	1.83149	1.55115	1.19287
C18	-0.16415	3.26648	0.21313
O19	0.97485	3.60602	-0.06762
C20	-1.16327	2.87193	-0.84574
H21	-0.70258	2.88050	-1.83295
H22	-1.56731	1.88251	-0.61371
H23	-2.00590	3.57115	-0.82670
C24	-0.65336	3.22809	1.64095
H25	-1.44336	3.97575	1.76915
H26	-1.09797	2.25271	1.85407
H27	0.16233	3.43871	2.33186
S28	-2.89707	-1.06982	0.95730
C29	-2.22595	-2.14365	-0.35650
H30	-1.17797	-2.36000	-0.14818
H31	-2.81630	-3.06034	-0.38964
H32	-2.32982	-1.59735	-1.29315
C33	-2.42825	-2.10554	2.38234
H34	-2.73107	-1.56805	3.27999
H35	-2.96028	-3.05538	2.31534
H36	-1.34826	-2.25618	2.36896
O37	-1.97740	0.16504	1.01233

Zero-point correction=	0.309537 (Hartree/Particle)
Thermal correction to Energy=	0.331147
Thermal correction to Enthalpy=	0.332091
Thermal correction to Gibbs Free Energy=	0.254836
Sum of electronic and zero-point Energies=	–1147.539532
Sum of electronic and thermal Energies	–1147.517922
Sum of electronic and thermal Enthalpies=	–1147.516978
Sum of electronic and thermal Free Energies=	–1147.594233

4A_GMS

Atom	X	Y	Z
C1	-1.5730555951	-2.0617530106	1.0844074249
C2	-2.7323730260	-2.2802811089	0.1059728514
C3	-2.3164806578	-1.4302581202	-1.1037891093
C4	-1.6661149450	-0.1976995567	-0.4448948324
H5	-0.1637807932	-0.5259396441	0.9594234732
H6	-1.8558333572	-2.2330990969	2.1249901679
H7	-0.7413167040	-2.7355054766	0.8444319623
H8	-2.8792464753	-3.3329704170	-0.1393404173
H9	-3.6655661460	-1.8999517990	0.5336682649
H10	-1.5684241260	-1.9600854394	-1.6994382920
H11	-3.1421550148	-1.1610515052	-1.7628008742
H12	-0.8638009587	0.2291581207	-1.0492739705
N13	-1.1762819740	-0.6454714362	0.8782011431
C14	-2.6738021053	0.9434113501	-0.2322984001
O15	-3.2719451558	1.4994021787	-1.1304087047
O16	-2.8195801686	1.2733042289	1.0493236647
H17	-2.1956741891	0.6187369928	1.5021290251
C18	1.2280891891	2.4388546489	-0.0957433584
O19	0.6566427911	2.1383023906	-1.1323870103
C20	0.4761623388	2.6663897142	1.1903770226
H21	-0.5933547517	2.7548604854	1.0066075310
H22	0.6644060460	1.8006993091	1.8326562159
H23	0.8492391112	3.5518461492	1.7111250571
C24	2.7270254156	2.6015777411	-0.0354388451
H25	2.9626822979	3.6626673805	0.0991446617
H26	3.1281137152	2.0652455031	0.8270968238
H27	3.1920239628	2.2450527401	-0.9541826025
S28	2.7064576826	-0.9788290882	-0.0299266417
C29	2.7444119928	-2.7687684100	0.3123174289
H30	1.7219947806	-3.1472855999	0.3338677877
H31	3.3317369206	-3.2605570519	-0.4642771678
H32	3.2225476968	-2.8977355312	1.2825116641
C33	1.8268701045	-1.0455717109	-1.6267607235
H34	1.5971423722	-0.0157020399	-1.8971981544
H35	2.4892931394	-1.4975512237	-2.3660661779
H36	0.9137745799	-1.6298966750	-1.5139535127
O37	1.7162380066	-0.3702299921	0.9810116246

Zero-point correction=	0.309634 (Hartree/Particle)
Thermal correction to Energy=	0.331102
Thermal correction to Enthalpy=	0.332046
Thermal correction to Gibbs Free Energy=	0.256055
Sum of electronic and zero-point Energies=	–1147.540681
Sum of electronic and thermal Energies=	–1147.519213
Sum of electronic and thermal Enthalpies=	–1147.518269
Sum of electronic and thermal Free Energies=	–1147.594260

5A_TS

Atom	X	Y	Z
C1	1.07268	0.23314	-0.98043
C2	2.04672	-0.93550	-0.69372
C3	1.81017	-1.26882	0.78917
C4	1.42844	0.10167	1.38810
H5	-0.38592	0.29634	0.50321
H6	1.57374	1.07633	-1.46070
H7	0.23062	-0.06498	-1.60463
H8	1.86419	-1.79020	-1.34582
H9	3.08001	-0.61539	-0.84909
H10	0.96226	-1.94809	0.90188
H11	2.67150	-1.71336	1.28528
H12	0.85463	0.00136	2.31038
N13	0.55338	0.68535	0.33993
C14	2.71046	0.87196	1.77256
O15	3.45474	0.35451	2.58995
O16	3.00434	2.03144	1.22657
H17	2.26322	2.51987	0.65827
C18	0.15875	2.65031	0.30573
O19	1.25546	3.17577	-0.08763
C20	-0.99196	2.63387	-0.68162
H21	-0.63278	2.37271	-1.67726
H22	-1.78659	1.95369	-0.37451
H23	-1.39636	3.65071	-0.72230
C24	-0.25033	2.80180	1.76181
H25	-0.65050	3.81387	1.88305
H26	-1.02550	2.08671	2.03962
H27	0.60738	2.70123	2.42733
S28	-2.91309	-1.39448	0.87631
C29	-2.37075	-2.22573	-0.65382
H30	-1.29499	-2.39690	-0.61640
H31	-2.91342	-3.16767	-0.74490
H32	-2.62832	-1.56351	-1.47934
C33	-2.20943	-2.57868	2.06931
H34	-2.41905	-2.18893	3.06447
H35	-2.69800	-3.54436	1.93304
H36	-1.13445	-2.65395	1.90450
O37	-2.06226	-0.11471	0.99773

Zero-point correction= 0.310786 (Hartree/Particle)
Thermal correction to Energy= 0.329901
Thermal correction to Enthalpy= 0.330846
Thermal correction to Gibbs Free Energy= 0.262461
Sum of electronic and zero-point Energies= –1147.525664
Sum of electronic and thermal Energies= –1147.506549
Sum of electronic and thermal Enthalpies= –1147.505605
Sum of electronic and thermal Free Energies= –1147.573989

5A_TS-1

Atom	X	Y	Z
C1	1.55009	-0.61757	-1.80702
C2	1.41179	-2.14894	-1.62180
C3	0.76510	-2.32501	-0.22299
C4	0.88552	-0.95108	0.44744
H5	-0.24236	-0.06215	-0.94799
H6	2.58859	-0.30508	-1.69749
H7	1.18040	-0.25102	-2.76384
H8	0.78597	-2.58739	-2.40002
H9	2.39128	-2.62567	-1.67422
H10	-0.29619	-2.55576	-0.32288
H11	1.24225	-3.10331	0.36936
H12	0.06362	-0.74969	1.13227
N13	0.75824	-0.00425	-0.70626
C14	2.19243	-0.76581	1.25233
O15	3.04284	-1.63638	1.30910
O16	2.30281	0.36241	1.92397
H17	1.56269	1.06827	1.63060
C18	0.89989	1.85913	-0.27321
O19	0.61367	1.89970	0.98666
C20	2.33014	2.18183	-0.68817
H21	3.06016	1.61100	-0.11571
H22	2.49697	2.02810	-1.75447
H23	2.48614	3.24247	-0.46951
C24	-0.14246	2.43145	-1.21969
H25	-0.06612	3.52181	-1.17169
H26	0.02398	2.12386	-2.25353
H27	-1.14666	2.14652	-0.91074
S28	-3.12531	0.22077	-0.09914
C29	-4.39273	-1.03301	0.27078
H30	-3.90733	-1.89445	0.73084
H31	-5.13214	-0.59094	0.94010
H32	-4.85761	-1.30943	-0.67454
C33	-2.49274	0.45303	1.59411
H34	-1.60424	1.08284	1.51669
H35	-3.26472	0.94956	2.18367
H36	-2.24432	-0.52022	2.01989
O37	-2.01474	-0.52751	-0.86868

Zero-point correction= 0.310951 (Hartree/Particle)
Thermal correction to Energy= 0.330107
Thermal correction to Enthalpy= 0.331051
Thermal correction to Gibbs Free Energy= 0.262038
Sum of electronic and zero-point Energies= –1147.522468
Sum of electronic and thermal Energies= –1147.503312
Sum of electronic and thermal Enthalpies= –1147.502368
Sum of electronic and thermal Free Energies= –1147.571381

5A_eq

Atom	X	Y	Z
C1	0.89099	-0.22200	-1.55635
C2	1.79950	-1.36382	-1.06599
C3	1.52664	-1.45515	0.44410
C4	1.28900	0.00552	0.84925
H5	-0.52126	0.02334	-0.05005
H6	1.41389	0.51329	-2.16055
H7	0.02107	-0.57725	-2.10529
H8	1.58650	-2.29803	-1.58534
H9	2.84584	-1.10949	-1.24444
H10	0.62473	-2.03978	0.63981
H11	2.34820	-1.88922	1.00998
H12	0.70302	0.08863	1.76235
N13	0.39388	0.46783	-0.29978
C14	2.63698	0.74708	1.07714
O15	3.26601	0.32862	2.06533
O16	2.99642	1.66157	0.28054
H17	1.91009	2.40289	-0.62498
C18	0.03707	1.97841	-0.47665
O19	1.07617	2.63367	-1.12551
C20	-1.19741	2.04461	-1.37185
H21	-0.98844	1.61913	-2.35415
H22	-2.03882	1.52137	-0.91712
H23	-1.46076	3.09446	-1.50580
C24	-0.24933	2.54799	0.91372
H25	-0.61650	3.56784	0.79190
H26	-1.01152	1.95990	1.42923
H27	0.65527	2.58439	1.52127
S28	-3.01424	-1.56969	0.24840
C29	-2.67441	-2.03303	-1.48077
H30	-1.64719	-2.38871	-1.56523
H31	-3.38105	-2.81078	-1.77288
H32	-2.83125	-1.14071	-2.08519
C33	-2.50143	-3.12870	1.03660
H34	-2.57366	-2.97713	2.11265
H35	-3.18704	-3.91645	0.72146
H36	-1.47664	-3.35842	0.74508
O37	-1.93545	-0.53094	0.63274

Zero-point correction=	0.315316 (Hartree/Particle)
Thermal correction to Energy=	0.334225
Thermal correction to Enthalpy=	0.335170
Thermal correction to Gibbs Free Energy=	0.267392
Sum of electronic and zero-point Energies=	–1147.538477
Sum of electronic and thermal Energies=	–1147.519567
Sum of electronic and thermal Enthalpies=	–1147.518623
Sum of electronic and thermal Free Energies=	–1147.586400

5A_pre-org

Atom	X	Y	Z
C1	1.65157	0.41531	-1.75691
C2	3.07014	0.48379	-1.19813
C3	2.95171	-0.11557	0.22466
C4	1.44323	-0.32131	0.46607
H5	-0.17210	0.15670	-0.77577
H6	1.42066	1.14229	-2.52747
H7	1.42290	-0.57736	-2.14837
H8	3.76297	-0.06618	-1.83522
H9	3.40334	1.52049	-1.14570
H10	3.46307	-1.07469	0.31449
H11	3.36593	0.55995	0.97082
H12	1.14500	-1.31531	0.13596
H13	0.75395	0.59623	-0.55586
C14	0.99750	-0.19905	1.95711
O15	0.35320	-1.16393	2.41095
O16	1.35698	0.83784	2.58612
H17	1.55498	2.04599	1.48622
C18	0.45225	2.07659	-0.11303
O19	1.52224	2.55893	0.63139
C20	0.30295	2.96383	-1.34301
H21	1.26440	3.13281	-1.82772
H22	-0.40607	2.54643	-2.06001
H23	-0.07760	3.92953	-1.00784
C24	-0.87165	2.03383	0.66293
H25	-1.06197	3.03433	1.05327
H26	-1.69152	1.75257	0.00017
H27	-0.84402	1.34165	1.50156
C28	-3.83921	-2.03928	-1.23354
H29	-3.35962	-2.73645	-1.92100
H30	-4.56081	-2.54782	-0.59318
H31	-4.32258	-1.22828	-1.77642
C32	-1.85322	-2.80844	0.52186
H33	-1.01593	-2.49241	1.14630
H34	-2.61813	-3.29729	1.12731
H35	-1.52500	-3.45200	-0.29550
O36	-1.49860	-0.75741	-1.17492
S37	-2.56237	-1.28933	-0.17917

Zero-point correction=	0.315264 (Hartree/Particle)
Thermal correction to Energy=	0.334190
Thermal correction to Enthalpy=	0.335134
Thermal correction to Gibbs Free Energy=	0.267268
Sum of electronic and zero-point Energies=	–1147.531521
Sum of electronic and thermal Energies=	–1147.512595
Sum of electronic and thermal Enthalpies=	–1147.511651
Sum of electronic and thermal Free Energies=	–1147.579517

6A_TS

Atom	X	Y	Z
C1	-0.38104	0.31631	-1.86408
C2	0.83867	-0.29283	-2.64219
C3	1.82895	-0.82918	-1.55058
C4	0.91103	-0.80667	-0.34610
H5	-0.36896	0.11462	0.71704
H6	-0.72662	1.26820	-2.25910
H7	-1.21657	-0.38165	-1.84780
H8	0.49166	-1.10616	-3.28089
H9	1.31682	0.44668	-3.28389
H10	2.17869	-1.83668	-1.77475
H11	2.70013	-0.18665	-1.42612
H12	0.15067	-1.58007	-0.54319
N13	0.10084	0.45103	-0.45593
C14	1.10641	-1.02004	1.14718
O15	1.97702	-1.68517	1.68156
O16	0.06389	-0.46729	1.74421
H17	1.29920	1.46182	1.71324
C18	0.77973	1.81139	-0.14668
O19	1.74389	1.62987	0.87202
C20	1.54669	2.44995	-1.30543
H21	2.40397	1.84930	-1.60042
H22	0.91434	2.63105	-2.17252
H23	1.91719	3.41354	-0.95304
C24	-0.35677	2.72259	0.32199
H25	0.04185	3.70877	0.56426
H26	-1.11481	2.83397	-0.45554
H27	-0.83786	2.30830	1.21171
S28	-2.72084	-2.92981	0.21818
C29	-1.76361	-3.47248	1.67490
H30	-1.05973	-2.68681	1.95095
H31	-2.46164	-3.68052	2.48719
H32	-1.24238	-4.38487	1.38739
C33	-3.32397	-1.35231	0.91119
H34	-3.85534	-0.83645	0.11239
H35	-4.00516	-1.57373	1.73413
H36	-2.47104	-0.76813	1.25531
O37	-1.69042	-2.55862	-0.86301

Zero-point correction=	0.309900 (Hartree/Particle)
Thermal correction to Energy=	0.328863
Thermal correction to Enthalpy=	0.329807
Thermal correction to Gibbs Free Energy=	0.261574
Sum of electronic and zero-point Energies=	–1147.488638
Sum of electronic and thermal Energies=	–1147.469676
Sum of electronic and thermal Enthalpies=	–1147.468731
Sum of electronic and thermal Free Energies=	–1147.536964

6A_eq

Atom	X	Y	Z
C1	0.86269	0.55144	-1.87803
C2	2.35100	0.12516	-1.74814
C3	2.54000	-0.15573	-0.23747
C4	1.12121	0.02542	0.34332
H5	-0.72993	-0.44874	1.87319
H6	0.70311	1.32602	-2.62339
H7	0.25894	-0.31269	-2.17377
H8	2.55743	-0.75402	-2.36164
H9	3.01890	0.92041	-2.08415
H10	2.91374	-1.16185	-0.03678
H11	3.23173	0.54962	0.22096
H12	0.58391	-0.91947	0.21232
N13	0.40341	0.97998	-0.54075
C14	1.07620	0.31003	1.84862
O15	2.02155	0.74885	2.47184
O16	-0.04152	-0.04586	2.48130
H17	-1.03348	2.15317	0.99488
C18	0.45833	2.42677	-0.23103
O19	-0.17380	2.59129	1.04618
C20	1.85508	3.03951	-0.06942
H21	2.38661	2.57123	0.75742
H22	2.43115	2.91438	-0.98731
H23	1.76551	4.10859	0.13644
C24	-0.34858	3.20379	-1.28129
H25	-0.51755	4.21255	-0.90114
H26	0.17496	3.28795	-2.23466
H27	-1.31450	2.72172	-1.45056
S28	-3.97898	-2.60849	0.31000
C29	-4.13632	-1.91966	-0.52045
H30	-4.24009	-3.63065	0.03320
H31	-4.54740	-2.29823	1.18547
H32	-1.51424	-3.00700	-0.84222
C33	-0.43064	-2.99471	-0.73360
H34	-1.85037	-4.00993	-1.10865
H35	-1.83779	-2.27037	-1.57825
H36	-2.22262	-2.56946	0.77696
O37	-1.90193	-1.06635	0.97013

Zero-point correction= 0.312731 (Hartree/Particle)
Thermal correction to Energy= 0.332228
Thermal correction to Enthalpy= 0.333172
Thermal correction to Gibbs Free Energy= 0.263394
Sum of electronic and zero-point Energies= –1147.517269
Sum of electronic and thermal Energies= –1147.497771
Sum of electronic and thermal Enthalpies= –1147.496827
Sum of electronic and thermal Free Energies= –1147.566605

4B_inp

Atom	X	Y	Z
C1	-2.72417	0.87076	-1.98040
C2	-2.36016	1.72826	-0.75467
C3	-1.19393	0.95218	-0.10520
C4	-1.31449	-0.49503	-0.72413
H5	-3.32179	-0.77840	-0.96782
H6	-3.75065	1.01000	-2.32103
H7	-2.05134	1.09237	-2.81704
H8	-2.08657	2.75421	-1.01122
H9	-3.21565	1.76615	-0.07478
H10	-0.22786	1.38497	-0.36778
H11	-1.26472	0.94219	0.98409
H12	-0.44015	-0.67116	-1.35782
N13	-2.52388	-0.52354	-1.55181
C14	-1.33791	-1.56996	0.34825
O15	-2.34603	-2.14318	0.70497
O16	-0.16501	-1.84508	0.92951
H17	0.58694	-1.33309	0.53078
C18	2.51316	0.39655	0.37816
O19	1.90063	-0.52561	-0.15208
C20	2.13040	0.92512	1.73312
H21	1.70863	1.92817	1.60904
H22	1.39577	0.28303	2.21640
H23	3.01624	1.02958	2.36428
C24	3.67621	1.04633	-0.31544
H25	3.57386	2.13443	-0.29029
H26	4.59309	0.80173	0.23134
H27	3.75908	0.69409	-1.34234
S28	-5.97773	-2.00033	-1.65790
C29	-5.22360	-1.76997	-3.30479
H30	-5.44522	-2.64673	-3.91479
H31	-5.68551	-0.88489	-3.74121
H32	-4.15010	-1.62470	-3.17852
C33	-4.98249	-3.44105	-1.14029
H34	-5.31738	-3.71474	-0.14041
H35	-5.16686	-4.26109	-1.83554
H36	-3.93134	-3.15345	-1.12064
O37	-5.49798	-0.81732	-0.79776

Zero-point correction=	0.309466 (Hartree/Particle)
Thermal correction to Energy=	0.331329
Thermal correction to Enthalpy=	0.332273
Thermal correction to Gibbs Free Energy=	0.253610
Sum of electronic and zero-point Energies=	–1147.531381
Sum of electronic and thermal Energies=	–1147.509518
Sum of electronic and thermal Enthalpies=	–1147.508574
Sum of electronic and thermal Free Energies=	–1147.587237

4B_LM-1

Atom	X	Y	Z
C1	0.05382	1.90358	-1.38113
C2	0.03264	1.71113	-2.92604
C3	-0.26984	0.20228	-3.12702
C4	-0.65127	-0.27984	-1.71634
H5	1.09655	0.24935	-0.77232
H6	0.89293	2.50730	-1.03139
H7	-0.86929	2.38860	-1.04647
H8	-0.72773	2.34330	-3.38954
H9	0.99425	1.97665	-3.36972
H10	-1.06387	0.01749	-3.85330
H11	0.62054	-0.33666	-3.45582
H12	-1.71476	-0.08860	-1.53289
N13	0.12053	0.56021	-0.77756
C14	-0.36877	-1.75025	-1.41979
O15	0.47366	-2.40389	-1.99673
O16	-1.07912	-2.29425	-0.42673
H17	-1.76495	-1.67633	-0.06212
C18	-3.22002	0.04681	1.35944
O19	-3.13120	-0.88467	0.56400
C20	-4.56008	0.49608	1.86735
H21	-4.76108	1.50201	1.48353
H22	-5.34792	-0.18404	1.54717
H23	-4.53998	0.57456	2.95807
C24	-1.99928	0.76246	1.86591
H25	-1.19579	0.71195	1.12706
H26	-2.22192	1.79678	2.13295
H27	-1.67180	0.25124	2.77912
S28	2.87183	-0.28723	1.48011
C29	2.08198	1.25686	2.05234
H30	1.91929	1.18726	3.12867
H31	2.77303	2.06872	1.82890
H32	1.14340	1.38498	1.51372
C33	1.49446	-1.41451	1.88587
H34	1.77988	-2.40478	1.53336
H35	1.36118	-1.42361	2.968502
H36	0.595556	-1.0691	1.377331
O37	2.914287	-0.20071	-0.05824

Zero-point correction=	0.309697 (Hartree/Particle)
Thermal correction to Energy=	0.331437
Thermal correction to Enthalpy=	0.332382
Thermal correction to Gibbs Free Energy=	0.254900
Sum of electronic and zero-point Energies=	–1147.531731
Sum of electronic and thermal Energies=	–1147.509990
Sum of electronic and thermal Enthalpies=	–1147.509046
Sum of electronic and thermal Free Energies=	–1147.586527

4B_LM-2

Atom	X	Y	Z
C1	-0.85602	1.84018	-1.69646
C2	-0.09542	1.44867	-2.97464
C3	-0.39147	-0.06232	-3.11382
C4	-0.86584	-0.49844	-1.69976
H5	0.18058	0.69559	-0.36931
H6	-0.46061	2.72867	-1.20080
H7	-1.91443	2.01894	-1.92024
H8	-0.40470	2.02384	-3.85013
H9	0.97496	1.61214	-2.82259
H10	-1.18263	-0.24814	-3.84319
H11	0.48784	-0.62695	-3.42312
H12	-1.92534	-0.77869	-1.73367
N13	-0.74714	0.67618	-0.80332
C14	-0.11267	-1.68611	-1.09974
O15	0.91381	-2.14564	-1.55253
O16	-0.64423	-2.20886	0.01237
H17	-1.49308	-1.76171	0.27222
C18	-2.63102	0.26121	1.01957
O19	-2.87826	-0.87441	0.60678
C20	-3.47235	1.43071	0.58921
H21	-2.87751	2.34096	0.51431
H22	-3.96085	1.21639	-0.36075
H23	-4.24232	1.59565	1.35211
C24	-1.63186	0.49411	2.12273
H25	-1.04731	1.39525	1.94250
H26	-2.19067	0.63385	3.05595
H27	-0.97502	-0.36701	2.23701
S28	2.90071	0.35619	1.27735
C29	2.39795	0.92617	2.93566
H30	2.97473	0.37775	3.68171
H31	2.62881	1.98926	2.99121
H32	1.32827	0.75800	3.06285
C33	2.34080	-1.37105	1.43400
H34	2.48744	-1.84341	0.46391
H35	2.94759	-1.86136	2.19639
H36	1.28399	-1.38732	1.69787
O37	1.94271	1.03509	0.28147

Zero-point correction=	0.310372 (Hartree/Particle)
Thermal correction to Energy=	0.331455
Thermal correction to Enthalpy=	0.332399
Thermal correction to Gibbs Free Energy=	0.258740
Sum of electronic and zero-point Energies=	–1147.531703
Sum of electronic and thermal Energies=	–1147.510620
Sum of electronic and thermal Enthalpies=	–1147.509676
Sum of electronic and thermal Free Energies=	–1147.583335

4B_LM-3

Atom	X	Y	Z
C1	-0.81228	1.94107	-1.51629
C2	-0.18553	1.68373	-2.91137
C3	-0.16506	0.13785	-3.04559
C4	-0.77044	-0.36058	-1.70986
H5	0.38766	0.70244	-0.42911
H6	-0.38542	2.80340	-1.00111
H7	-1.89076	2.10973	-1.61361
H8	-0.75723	2.16501	-3.70774
H9	0.83073	2.08211	-2.94812
H10	-0.73812	-0.22082	-3.90203
H11	0.85332	-0.23832	-3.15033
H12	-1.85125	-0.51531	-1.84335
N13	-0.59354	0.72009	-0.72502
C14	-0.23098	-1.69940	-1.22897
O15	0.35867	-2.47756	-1.94687
O16	-0.47576	-2.03600	0.04630
H17	-0.95929	-1.35203	0.56352
C18	-2.25853	0.53193	1.66868
O19	-1.62208	-0.47242	1.97105
C20	-3.42002	0.47169	0.71589
H21	-3.40352	1.31868	0.02855
H22	-3.42274	-0.46725	0.16425
H23	-4.34437	0.54376	1.30058
C24	-1.94051	1.87038	2.27435
H25	-1.50966	2.49785	1.48771
H26	-2.85019	2.36574	2.62333
H27	-1.22504	1.76773	3.08874
S28	2.90333	0.00214	1.18893
C29	1.68907	0.45773	2.47157
H30	2.06790	0.12513	3.43919
H31	1.61010	1.54432	2.45597
H32	0.72648	-0.00102	2.24465
C33	2.74364	-1.80875	1.33226
H34	3.40498	-2.24481	0.58432
H35	3.06098	-2.10799	2.33225
H36	1.70753	-2.08670	1.13967
O37	2.25739	0.37984	-0.15928

Zero-point correction=	0.309541 (Hartree/Particle)
Thermal correction to Energy=	0.331144
Thermal correction to Enthalpy=	0.332088
Thermal correction to Gibbs Free Energy=	0.255922
Sum of electronic and zero-point Energies=	–1147.533389
Sum of electronic and thermal Energies=	–1147.511786
Sum of electronic and thermal Enthalpies=	–1147.510842
Sum of electronic and thermal Free Energies=	–1147.587008

4B_GMS

Atom	X	Y	Z
C1	-0.97824	1.13659	-1.82654
C2	0.21969	0.57721	-2.61281
C3	0.28537	-0.89988	-2.16143
C4	-0.55257	-0.94829	-0.85323
H5	-0.26212	0.88010	0.07075
H6	-0.93768	2.21597	-1.66864
H7	-1.91667	0.90507	-2.34447
H8	0.10831	0.68459	-3.69395
H9	1.12894	1.10599	-2.31530
H10	-0.15305	-1.56579	-2.90740
H11	1.30883	-1.22882	-1.98248
H12	-1.46542	-1.53307	-1.01837
N13	-0.96308	0.43878	-0.53216
C14	0.15969	-1.57166	0.34844
O15	1.34547	-1.83325	0.37595
O16	-0.59711	-1.80915	1.42213
H17	-1.55333	-1.57208	1.27620
C18	-3.22825	0.12772	0.82326
O19	-3.09849	-1.09767	0.88629
C20	-2.69536	1.02858	1.90574
H21	-3.52217	1.23935	2.59521
H22	-1.89517	0.53957	2.45928
H23	-2.34487	1.97632	1.49944
C24	-4.10566	0.74959	-0.22707
H25	-4.28207	0.04710	-1.04087
H26	-5.06567	1.00802	0.23497
H27	-3.66456	1.67083	-0.60826
S28	2.45276	1.75860	1.48797
C29	2.66160	0.35976	2.63974
H30	3.72866	0.20768	2.80896
H31	2.17358	0.64541	3.57105
H32	2.19802	-0.52727	2.20790
C33	3.22824	0.99180	0.02499
H34	3.11078	1.69641	-0.79737
H35	4.28776	0.84138	0.23769
H36	2.72909	0.04709	-0.18932
O37	0.94417	1.84081	1.18686

Zero-point correction= 0.310374 (Hartree/Particle)
Thermal correction to Energy= 0.331477
Thermal correction to Enthalpy= 0.332421
Thermal correction to Gibbs Free Energy= 0.258015
Sum of electronic and zero-point Energies= –1147.533811
Sum of electronic and thermal Energies= –1147.512707
Sum of electronic and thermal Enthalpies= –1147.511763
Sum of electronic and thermal Free Energies= –1147.586170

5B_TS

Atom	X	Y	Z
C1	-1.09395	1.21968	-1.62083
C2	0.10096	0.68837	-2.41269
C3	-0.01601	-0.83354	-2.21420
C4	-0.66495	-0.99889	-0.82305
H5	-0.42894	0.76166	0.24924
H6	-1.01658	2.26619	-1.32739
H7	-2.01942	1.07480	-2.18063
H8	0.07259	0.98510	-3.46221
H9	1.02923	1.06357	-1.97562
H10	-0.66862	-1.26793	-2.97393
H11	0.94551	-1.34244	-2.25922
H12	-1.54746	-1.64291	-0.86649
N13	-1.12500	0.36655	-0.40281
C14	0.24997	-1.58977	0.26502
O15	1.39738	-1.93814	0.04748
O16	-0.28952	-1.68910	1.46280
H17	-1.28782	-1.36459	1.46687
C18	-2.72531	0.23730	0.68444
O19	-2.63431	-0.92243	1.23896
C20	-2.49191	1.45627	1.56328
H21	-3.36846	1.56901	2.20915
H22	-1.61096	1.31119	2.18898
H23	-2.37384	2.37118	0.97962
C24	-3.82551	0.38406	-0.34998
H25	-3.75468	-0.40770	-1.09832
H26	-4.77716	0.26520	0.17627
H27	-3.82577	1.35892	-0.83797
S28	2.30401	1.62103	1.55873
C29	2.49444	0.16687	2.64177
H30	3.55255	0.06218	2.88632
H31	1.92154	0.37296	3.54513
H32	2.11351	-0.71660	2.13072
C33	3.20556	0.97471	0.11085
H34	3.13023	1.72971	-0.67078
H35	4.25030	0.83670	0.39347
H36	2.75303	0.03339	-0.19989
O37	0.81481	1.67086	1.16004

Zero-point correction=	0.311476 (Hartree/Particle)
Thermal correction to Energy=	0.330283
Thermal correction to Enthalpy=	0.331227
Thermal correction to Gibbs Free Energy=	0.264328
Sum of electronic and zero-point Energies=	–1147.528905
Sum of electronic and thermal Energies=	–1147.510098
Sum of electronic and thermal Enthalpies=	–1147.509154
Sum of electronic and thermal Free Energies=	–1147.576053

5B_eq

Atom	X	Y	Z
C1	-1.10418	1.23198	-1.77974
C2	-0.12330	0.74850	-2.83896
C3	-0.39094	-0.76148	-2.87441
C4	-0.62720	-1.14215	-1.40714
H5	-0.15728	0.47251	-0.15223
H6	-0.85960	2.19444	-1.33848
H7	-2.12267	1.24140	-2.16244
H8	-0.29207	1.23908	-3.79774
H9	0.90066	0.95588	-2.51757
H10	-1.28621	-0.96961	-3.46535
H11	0.43601	-1.33901	-3.28233
H12	-1.46863	-1.82388	-1.29184
N13	-0.99572	0.17190	-0.70636
C14	0.57229	-1.81078	-0.66439
O15	1.41867	-2.40684	-1.34607
O16	0.54795	-1.69962	0.60078
H17	-0.95921	-1.26116	1.11553
C18	-2.16272	0.09054	0.30933
O19	-1.92914	-1.02974	1.12127
C20	-2.14270	1.37686	1.13639
H21	-2.91196	1.29045	1.90481
H22	-1.17231	1.50521	1.61693
H23	-2.35836	2.25800	0.52947
C24	-3.49719	-0.11428	-0.39887
H25	-3.45716	-0.95443	-1.09380
H26	-4.23664	-0.34717	0.36831
H27	-3.82822	0.77866	-0.92854
S28	2.34671	1.44982	1.17733
C29	1.85367	0.49898	2.65050
H30	2.74706	0.29990	3.24439
H31	1.16355	1.12641	3.21350
H32	1.37519	-0.42065	2.31601
C33	3.37942	0.17766	0.38185
H34	3.67150	0.57516	-0.58956
H35	4.26324	0.02361	1.00290
H36	2.79973	-0.73802	0.27601
O37	1.07255	1.57740	0.31098

Zero-point correction=	0.314879 (Hartree/Particle)
Thermal correction to Energy=	0.333770
Thermal correction to Enthalpy=	0.334714
Thermal correction to Gibbs Free Energy=	0.267560
Sum of electronic and zero-point Energies=	–1147.541380
Sum of electronic and thermal Energies=	–1147.522489
Sum of electronic and thermal Enthalpies=	–1147.521545
Sum of electronic and thermal Free Energies=	–1147.588699

5B_1

Atom	X	Y	Z
C1	-0.45738	1.58977	-1.62299
C2	0.01940	0.94057	-2.91808
C3	-0.95327	-0.23325	-3.08530
C4	-1.08982	-0.78664	-1.65898
H5	-0.01258	0.15075	-0.21091
H6	0.29439	2.16149	-1.08667
H7	-1.33308	2.21283	-1.78946
H8	-0.00642	1.64386	-3.75071
H9	1.04477	0.57750	-2.80721
H10	-1.92126	0.12609	-3.44515
H11	-0.60081	-1.00515	-3.76766
H12	-2.07387	-1.20736	-1.47254
N13	-0.86095	0.41588	-0.75443
C14	-0.02755	-1.87791	-1.32150
O15	-0.14111	-2.94206	-1.96017
O16	0.82588	-1.56962	-0.44840
H17	-0.65565	1.78710	1.31659
C18	-1.94596	0.72910	0.31005
O19	-1.55590	1.91493	0.94470
C20	-3.29633	0.98275	-0.34338
H21	-4.00551	1.23268	0.44647
H22	-3.25236	1.82316	-1.03594
H23	-3.66826	0.10353	-0.86875
C24	-1.97885	-0.44533	1.28835
H25	-1.00027	-0.58190	1.74802
H26	-2.70460	-0.21587	2.06927
H27	-2.27428	-1.37778	0.80570
S28	2.23953	0.77629	1.97997
C29	1.73692	-0.85502	2.61615
H30	2.61383	-1.32682	3.06162
H31	0.98180	-0.67979	3.38163
H32	1.34254	-1.44011	1.78541
C33	3.38158	0.20039	0.68575
H34	3.73153	1.08719	0.15879
H35	4.21983	-0.30601	1.16658
H36	2.82849	-0.46626	0.02388
O37	1.02038	1.32654	1.19974

Zero-point correction=	0.315352 (Hartree/Particle)
Thermal correction to Energy=	0.334336
Thermal correction to Enthalpy=	0.335280
Thermal correction to Gibbs Free Energy=	0.268286
Sum of electronic and zero-point Energies=	–1147.543657
Sum of electronic and thermal Energies=	–1147.524673
Sum of electronic and thermal Enthalpies=	–1147.523729
Sum of electronic and thermal Free Energies=	–1147.590723

5B_2

Atom	X	Y	Z
C1	-1.06639	0.91108	-2.33231
C2	-0.59583	-0.06417	-3.41068
C3	-0.96864	-1.43054	-2.82146
C4	-0.58408	-1.28520	-1.34762
H5	0.09719	0.44966	-0.65476
H6	-0.52763	1.85551	-2.30633
H7	-2.13057	1.11322	-2.42977
H8	-1.07656	0.14103	-4.36727
H9	0.48571	0.01082	-3.54821
H10	-2.04311	-1.60862	-2.91841
H11	-0.43953	-2.26410	-3.28050
H12	-1.16311	-1.90975	-0.67373
N13	-0.83966	0.17877	-1.02236
C14	0.92587	-1.56391	-1.07362
O15	1.31402	-2.72402	-1.29757
O16	1.59397	-0.58023	-0.64561
H17	-0.69961	0.20979	1.70118
C18	-1.86178	0.44377	0.12331
O19	-1.40986	-0.28809	1.21796
C20	-1.86543	1.94773	0.39209
H21	-2.52892	2.13898	1.23652
H22	-0.86484	2.29166	0.66010
H23	-2.23018	2.51321	-0.46581
C24	-3.23536	-0.08853	-0.26415
H25	-3.20677	-1.16500	-0.43588
H26	-3.91081	0.10342	0.56997
H27	-3.63886	0.40464	-1.14809
S28	1.85899	0.93501	2.92571
C29	2.22493	-0.82403	2.62682
H30	3.29018	-0.98170	2.80266
H31	1.63925	-1.39364	3.34741
H32	1.95292	-1.06700	1.59945
C33	2.77378	1.63052	1.51316
H34	2.57579	2.70178	1.50850
H35	3.83757	1.44831	1.67355
H36	2.42192	1.15043	0.60069
O37	0.36024	1.12471	2.58552

Zero-point correction=	0.315056 (Hartree/Particle)
Thermal correction to Energy=	0.334065
Thermal correction to Enthalpy=	0.335010
Thermal correction to Gibbs Free Energy=	0.267311
Sum of electronic and zero-point Energies=	–1147.542199
Sum of electronic and thermal Energies=	–1147.523189
Sum of electronic and thermal Enthalpies=	–1147.522245
Sum of electronic and thermal Free Energies=	–1147.589943

6B_TS

Atom	X	Y	Z
C1	-0.41123	1.50688	-1.06529
C2	0.27249	0.77192	-2.21964
C3	-0.74692	-0.31481	-2.58983
C4	-1.28804	-0.75873	-1.22424
H5	-0.27642	-0.29332	0.42488
H6	0.28115	1.99718	-0.38459
H7	-1.12933	2.23536	-1.45014
H8	0.50597	1.44213	-3.04739
H9	1.20718	0.31847	-1.87906
H10	-1.55970	0.11251	-3.18280
H11	-0.32007	-1.14892	-3.14710
H12	-2.31468	-1.11492	-1.26081
N13	-1.12594	0.42504	-0.31834
C14	-0.42290	-1.85849	-0.55842
O15	-0.33344	-2.98964	-1.01539
O16	0.17338	-1.39825	0.50376
H17	-2.11366	-0.53901	1.87941
C18	-2.33476	0.82479	0.48009
O19	-2.75860	-0.33635	1.18961
C20	-3.51472	1.22854	-0.40036
H21	-3.25940	2.07795	-1.03428
H22	-3.83766	0.40051	-1.03243
H23	-4.35056	1.51639	0.23881
C24	-1.93774	1.93653	1.44912
H25	-1.71273	2.86295	0.91808
H26	-2.77344	2.12215	2.12563
H27	-1.05233	1.64864	2.01866
S28	2.65273	0.81443	1.97895
C29	2.37191	-0.71214	2.94051
H30	3.32515	-1.22931	3.06074
H31	1.98743	-0.40707	3.91328
H32	1.64645	-1.32460	2.40566
C33	3.22006	0.01756	0.43636
H34	3.33824	0.81081	-0.30101
H35	4.18107	-0.46245	0.62675
H36	2.46755	-0.70428	0.12120
O37	1.26125	1.39492	1.67939

Zero-point correction=	0.309621 (Hartree/Particle)
Thermal correction to Energy=	0.328759
Thermal correction to Enthalpy=	0.329703
Thermal correction to Gibbs Free Energy=	0.262021
Sum of electronic and zero-point Energies	–1147.528246
Sum of electronic and thermal Energies=	–1147.509108
Sum of electronic and thermal Enthalpies=–	–1147.508164
Sum of electronic and thermal Free Energies=–	–1147.575847

6B_TS-1

Atom	X	Y	Z
C1	-0.04769	1.91159	-1.13164
C2	0.26713	1.35181	-2.51987
C3	-1.00233	0.56073	-2.86400
C4	-1.37165	-0.08980	-1.52252
H5	-0.00269	-0.18268	-0.07925
H6	0.83328	2.13240	-0.53307
H7	-0.65670	2.81420	-1.20875
H8	0.48800	2.14162	-3.23840
H9	1.13214	0.68369	-2.47455
H10	-1.80257	1.23948	-3.16991
H11	-0.86430	-0.18156	-3.65039
H12	-2.44162	-0.26176	-1.42260
N13	-0.82386	0.81921	-0.46602
C14	-0.62760	-1.43156	-1.28909
O15	-0.85316	-2.43377	-1.95108
O16	0.24418	-1.31198	-0.32769
H17	-0.15719	1.63587	1.72704
C18	-1.74669	1.25051	0.64845
O19	-0.99847	2.09101	1.50071
C20	-2.22055	0.00679	1.40996
H21	-2.80663	-0.66801	0.78300
H22	-1.36695	-0.54093	1.80945
H23	-2.84742	0.32968	2.24205
C24	-2.92955	2.07192	0.13883
H25	-3.56150	1.48693	-0.53107
H26	-3.53367	2.38440	0.99223
H27	-2.59121	2.96703	-0.38349
S28	2.30590	-0.07539	2.43741
C29	1.22103	-1.47030	2.88935
H30	1.83778	-2.24390	3.34923
H31	0.49994	-1.09013	3.61207
H32	0.72735	-1.83724	1.99006
C33	3.26177	-0.92057	1.13744
H34	3.94616	-0.18305	0.72012
H35	3.82259	-1.73731	1.59399
H36	2.56398	-1.28289	0.38281
O37	1.42054	0.93735	1.67898

Zero-point correction=	0.310072 (Hartree/Particle)
Thermal correction to Energy=	0.328754
Thermal correction to Enthalpy=	0.329699
Thermal correction to Gibbs Free Energy=	0.263446
Sum of electronic and zero-point Energies=	–1147.535622
Sum of electronic and thermal Energies=	–1147.516939
Sum of electronic and thermal Enthalpies=	–1147.515995
Sum of electronic and thermal Free Energies=	–1147.582247

6B_TS-2

Atom	X	Y	Z
C1	-1.25527	0.86650	-2.38637
C2	-0.66289	-0.05746	-3.45308
C3	-0.90762	-1.45516	-2.86679
C4	-0.62858	-1.25073	-1.37259
H5	0.33869	0.35534	-0.73684
H6	-0.82618	1.86786	-2.38932
H7	-2.33633	0.95185	-2.51591
H8	-1.13326	0.09032	-4.42554
H9	0.40930	0.12732	-3.56426
H10	-1.95044	-1.75092	-3.00737
H11	-0.27232	-2.22992	-3.29627
H12	-1.18511	-1.92530	-0.72658
N13	-0.94413	0.18844	-1.08845
C14	0.87404	-1.38137	-1.02027
O15	1.48155	-2.43939	-1.09925
O16	1.36343	-0.23212	-0.64661
H17	-0.62811	0.30037	1.59728
C18	-1.88082	0.44547	0.07243
O19	-1.34784	-0.23483	1.18544
C20	-1.92951	1.95507	0.31608
H21	-2.53181	2.14600	1.20574
H22	-0.92344	2.34236	0.48833
H23	-2.38068	2.48498	-0.52409
C24	-3.27266	-0.13545	-0.17640
H25	-3.22518	-1.21747	-0.30991
H26	-3.89690	0.07222	0.69387
H27	-3.74862	0.30774	-1.05176
S28	1.91302	0.92169	2.94189
C29	2.13009	-0.86225	2.63565
H30	3.14927	-1.13625	2.91183
H31	1.41734	-1.38061	3.27588
H32	1.93573	-1.06263	1.58314
C33	3.04936	1.53871	1.65836
H34	2.97162	2.62536	1.66686
H35	4.06368	1.23526	1.92261
H36	2.74513	1.12562	0.69743
O37	0.49027	1.26888	2.44529

Zero-point correction=	0.309846 (Hartree/Particle)
Thermal correction to Energy=	0.328654
Thermal correction to Enthalpy=	0.329598
Thermal correction to Gibbs Free Energy=	0.262280
Sum of electronic and zero-point Energies=	–1147.536777
Sum of electronic and thermal Energies=	–1147.517970
Sum of electronic and thermal Enthalpies=	–1147.517026
Sum of electronic and thermal Free Energies=	–1147.584344

6B_eq

Atom	X	Y	Z
C1	-1.33708	0.92993	-2.33358
C2	-0.65282	0.09730	-3.41878
C3	-0.85808	-1.33507	-2.91033
C4	-0.65467	-1.18178	-1.38955
H5	0.74018	0.33933	-0.47460
H6	-0.97214	1.95678	-2.28831
H7	-2.41840	0.96198	-2.51751
H8	-1.08390	0.26994	-4.40575
H9	0.41398	0.33639	-3.46856
H10	-1.88382	-1.66058	-3.10139
H11	-0.18045	-2.06745	-3.34921
H12	-1.23870	-1.90925	-0.82572
N13	-1.02721	0.21403	-1.07004
C14	0.80830	-1.43490	-1.00133
O15	1.35775	-2.50950	-1.12790
O16	1.44136	-0.36807	-0.51758
H17	-0.62536	0.33386	1.47552
C18	-1.97866	0.39578	0.06092
O19	-1.39572	-0.21280	1.20553
C20	-2.17680	1.89712	0.29961
H21	-2.76686	2.03150	1.20800
H22	-1.20979	2.38512	0.43317
H23	-2.70797	2.37297	-0.52664
C24	-3.32489	-0.30307	-0.15921
H25	-3.19778	-1.38315	-0.25090
H26	-3.97688	-0.11138	0.69494
H27	-3.81889	0.06930	-1.05838
S28	1.95425	1.11506	2.67443
C29	2.04107	-0.70311	2.79254
H30	2.95995	-0.97015	3.31610
H31	1.17488	-1.02437	3.36981
H32	2.01747	-1.11914	1.78642
C33	3.38543	1.36363	1.57435
H34	3.42847	2.42846	1.34866
H35	4.28541	1.05834	2.10996
H36	3.23796	0.77611	0.66928
O37	0.70521	1.42630	1.81646

Zero-point correction=	0.313339 (Hartree/Particle)
Thermal correction to Energy=	0.332540
Thermal correction to Enthalpy=	0.333484
Thermal correction to Gibbs Free Energy=	0.264890
Sum of electronic and zero-point Energies=	–1147.537170
Sum of electronic and thermal Energies=	–1147.517969
Sum of electronic and thermal Enthalpies=	–1147.517025
Sum of electronic and thermal Free Energies=	–1147.585618

End of Part 1

Part S2

Data pertaining to the 1st and 2nd proton transfers

Table S1. Energies (in a.u.) and associated changes (in kcal/mol) relative to GMS for 2-MC of proline (**1**) acetone (**2**) and 3-MC of proline (**1**) and acetone (**2**) and DMSO solvent molecule (**3**).

**Data for LEC**								
	E	**Δ*E***	***E*ZPVE**	**Δ*E*ZPVE**	***H***	**Δ*H***	***G***	**Δ*G***
	3-MC							
4A_GMS	-1147.8503	0.00	-1147.5407	0.00	-1147.5183	0.00	-1147.5943	0.00
4A_LM-3	-1147.8491	0.78	-1147.5395	0.72	-1147.5170	0.81	-1147.5942	0.02
5A_TS	-1147.8365	8.70	-1147.5257	9.42	-1147.5056	7.95	-1147.5740	12.72
5A_eq	-1147.8538	-2.18	-1147.5385	1.38	-1147.5186	-0.22	-1147.5864	4.93
5A_TS-1	-1147.8334	10.61	-1147.5225	11.43	-1147.5024	9.98	-1147.5714	14.36
5A_eq-1	-1147.8486	1.08	-1147.5334	4.60	-1147.5133	3.10	-1147.5813	8.15
5A_p-org	-1147.8468	2.22	-1147.5315	5.75	-1147.5117	4.15	-1147.5795	9.3
6A_TS	-1147.7985	32.49	-1147.4886	32.66	-1147.4687	31.09	-1147.5370	36.0
6A_eq	-1147.8300	12.75	-1147.5173	14.69	-1147.4968	13.46	-1147.5666	17.3
	2-MC							
4a_GMS	-594.5478	0.00	-594.3188	0.00	-594.3034	0.00	-594.3632	0.00
4a_p-org	-594.5467	0.65	-594.3182	0.38	-594.3026	0.51	-594.3637	-0.32
5a_TS	-594.5303	10.96	-594.3007	11.37	-594.2881	9.61	-594.3383	15.64
5a_eq	-594.5442	2.21	-594.3097	5.73	-594.2970	4.04	-594.3470	10.17
5a_p-org	-594.5368	6.86	-594.3018	10.67	-594.2892	8.92	-594.3388	15.30
6a_TS	-594.4998	30.12	-594.2700	30.60	-594.2578	28.63	-594.3061	35.85
6a_eq	-594.5231	15.46	-594.2906	17.73	-594.2774	16.34	-594.3279	22.11
**Data for HEC**								
	3-MC							
4B_GMS	-1147.8442	0.00	-1147.5338	0.00	-1147.5118	0.00	-1147.5862	0.00
5B_TS	-1147.8404	2.39	-1147.5289	3.08	-1147.5092	1.64	-1147.5761	6.35
5B_eq	-1147.8563	-7.58	-1147.5414	-4.75	-1147.5220	-6.14	-1147.5890	-1.59
5B_LM-1(GMS)	-1147.8590	-9.30	-1147.5437	-6.18	-1147.5237	-7.51	-1147.5907	-2.86
5B_LM-2	-1147.8573	-8.23	-1147.5422	-5.26	-1147.5222	-6.58	-1147.5899	-2.37
6B_TS	-1147.8379	3.96	-1147.5282	3.49	-1147.5082	2.26	-1147.5758	6.48
6B_LM-1-TS	-1147.8457	-0.95	-1147.5356	-1.14	-1147.5160	-2.66	-1147.5822	2.46
6B_LM_2-TS	-1147.8466	-1.52	-1147.5368	-1.86	-1147.5170	-3.30	-1147.5843	1.15
6B_eq	-1147.8505	-3.96	-1147.5372	-2.11	-1147.5170	-3.30	-1147.586	0.35
	2-MC							
4b_GMS	-594.5460	0.00	-594.3171	0.00	-594.3017	0.00	-594.3623	0.00
4b_p-org	-594.5428	2.00	-594.3136	2.18	-594.2985	2.00	-594.3564	3.72
5b_TS	-594.5336	7.82	-594.3029	8.91	-594.2904	7.10	-594.3404	13.78
5b_eq	-594.5479	-1.18	-594.3138	2.03	-594.3008	0.57	-594.3520	6.50
6b_TS	-594.5405	3.46	-594.3112	3.71	-594.2985	1.97	-594.3481	8.97
6b_eq	-594.5456	0.23	-594.3130	2.55	-594.3000	1.06	-594.3506	7.37

Table S2. Ball and stick models of 3-MC (Part A) and 2-MC (Part B) involving the LEC of proline. (For relevant energies – see Table S1).

Part A

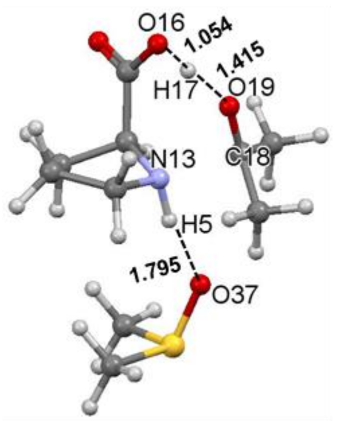 **5A_TS**	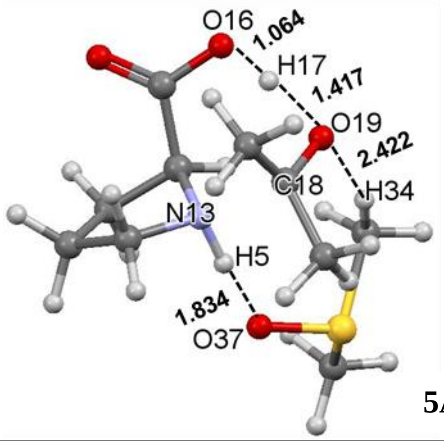 **5A_TS-1**
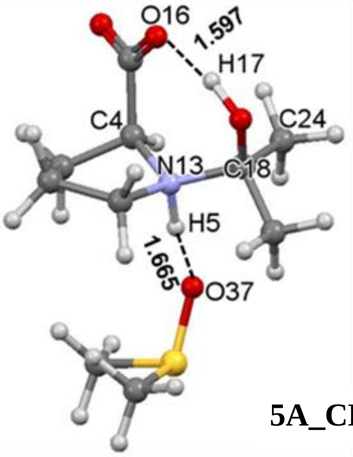 **5A_CN-eq**	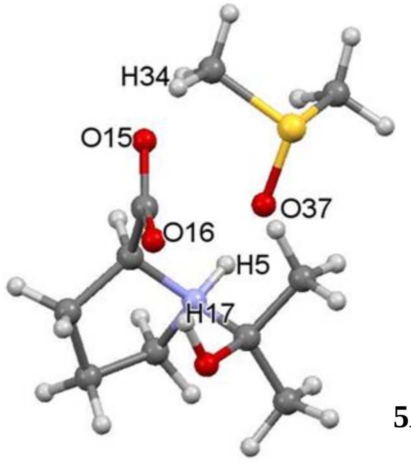 **5A_p-org**
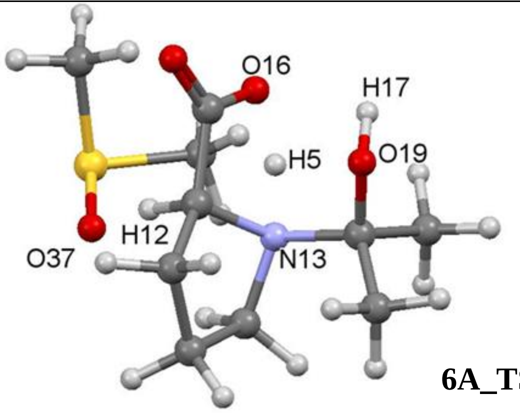 **6A_TS**	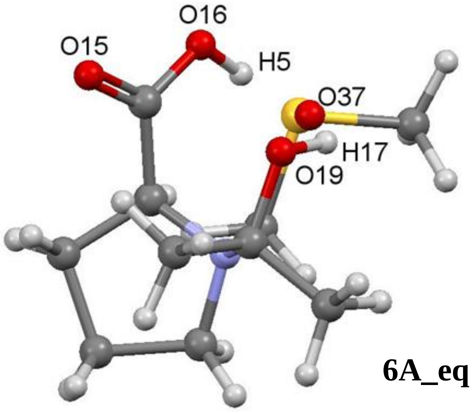 **6A_eq**

Part B

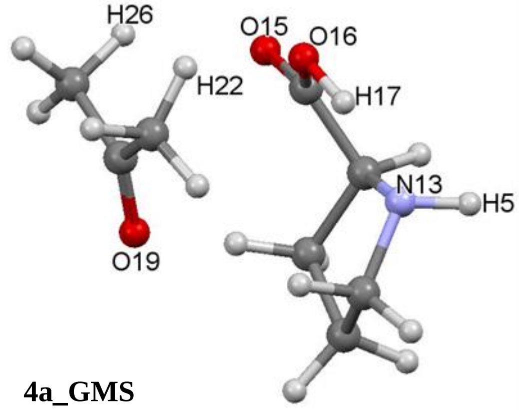

4a_GMS

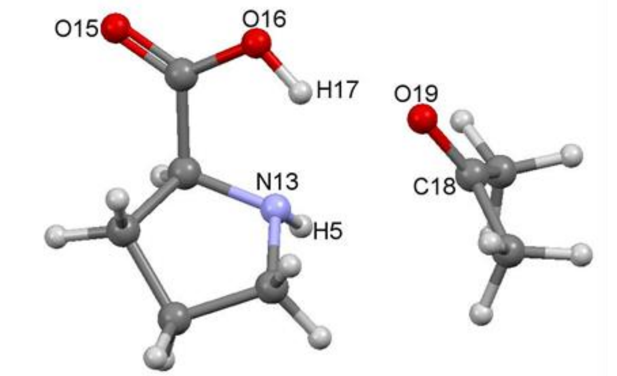

4a_p-org

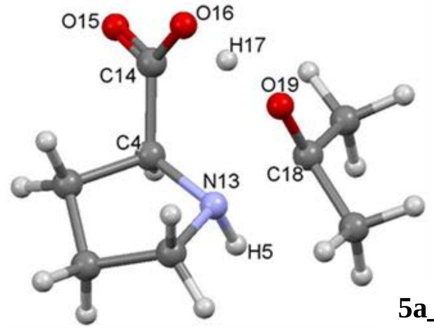

5a_TS

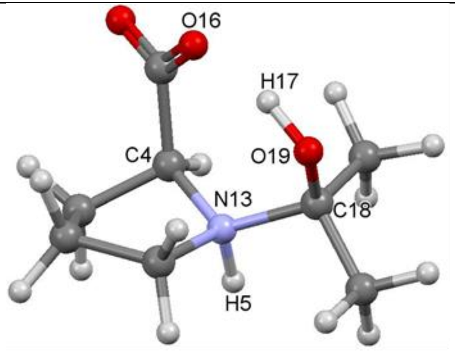

5a_eq

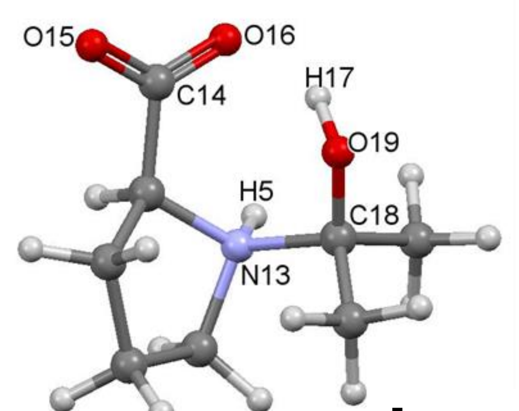

5a_p-org

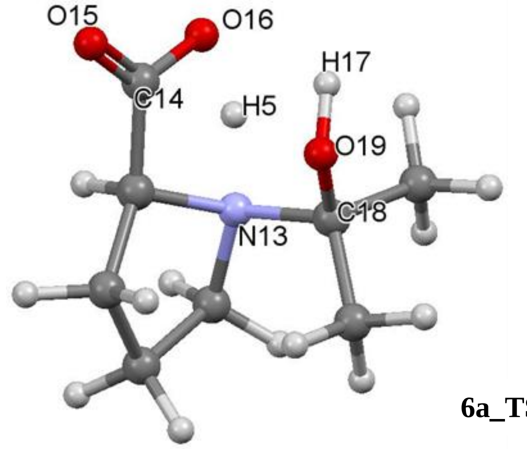

6a_TS

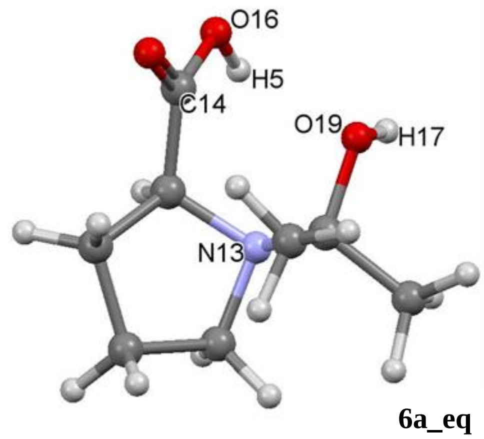

6a_eq

Table S3. Ball and stick models of 3-MC (Part A) and 2-MC (Part B) involving the HEC of proline. (For relevant energies – see Table S1).

Part A

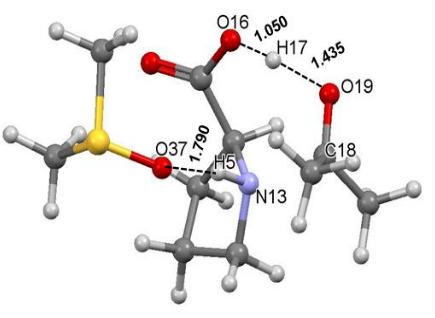

5B_TS

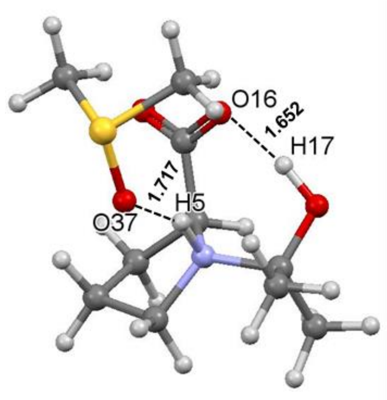

5B_eq

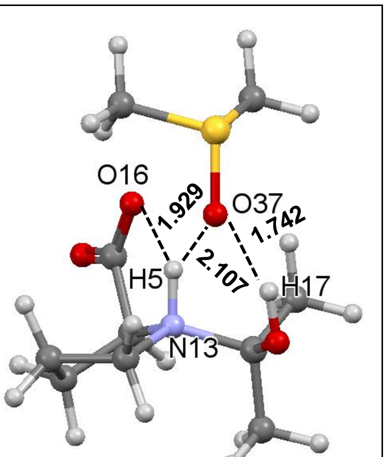

5B_LM-1 (GMS)

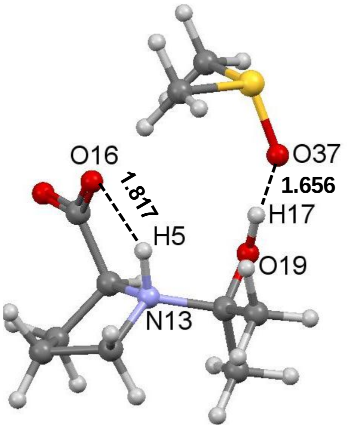

5B_LM-2

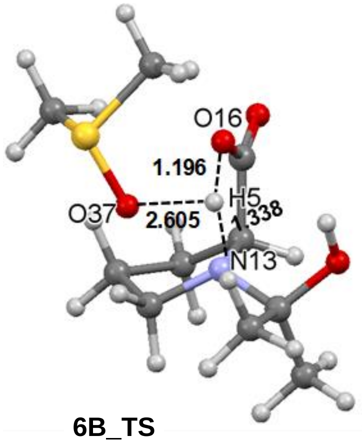

6B_TS

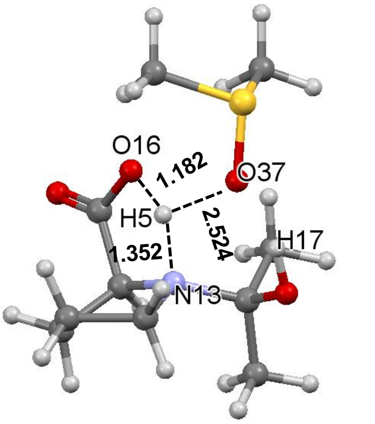

6B_LM-1-TS

6B_LM-2-TS

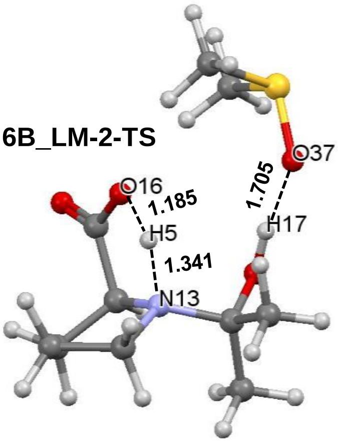

6B_eq

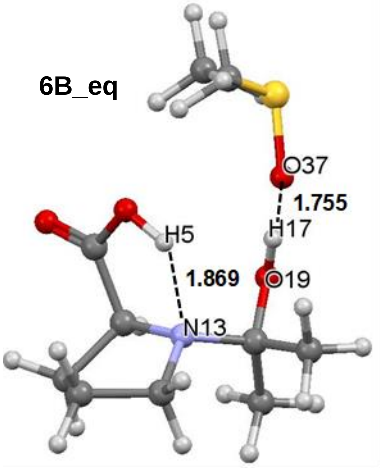

Part B

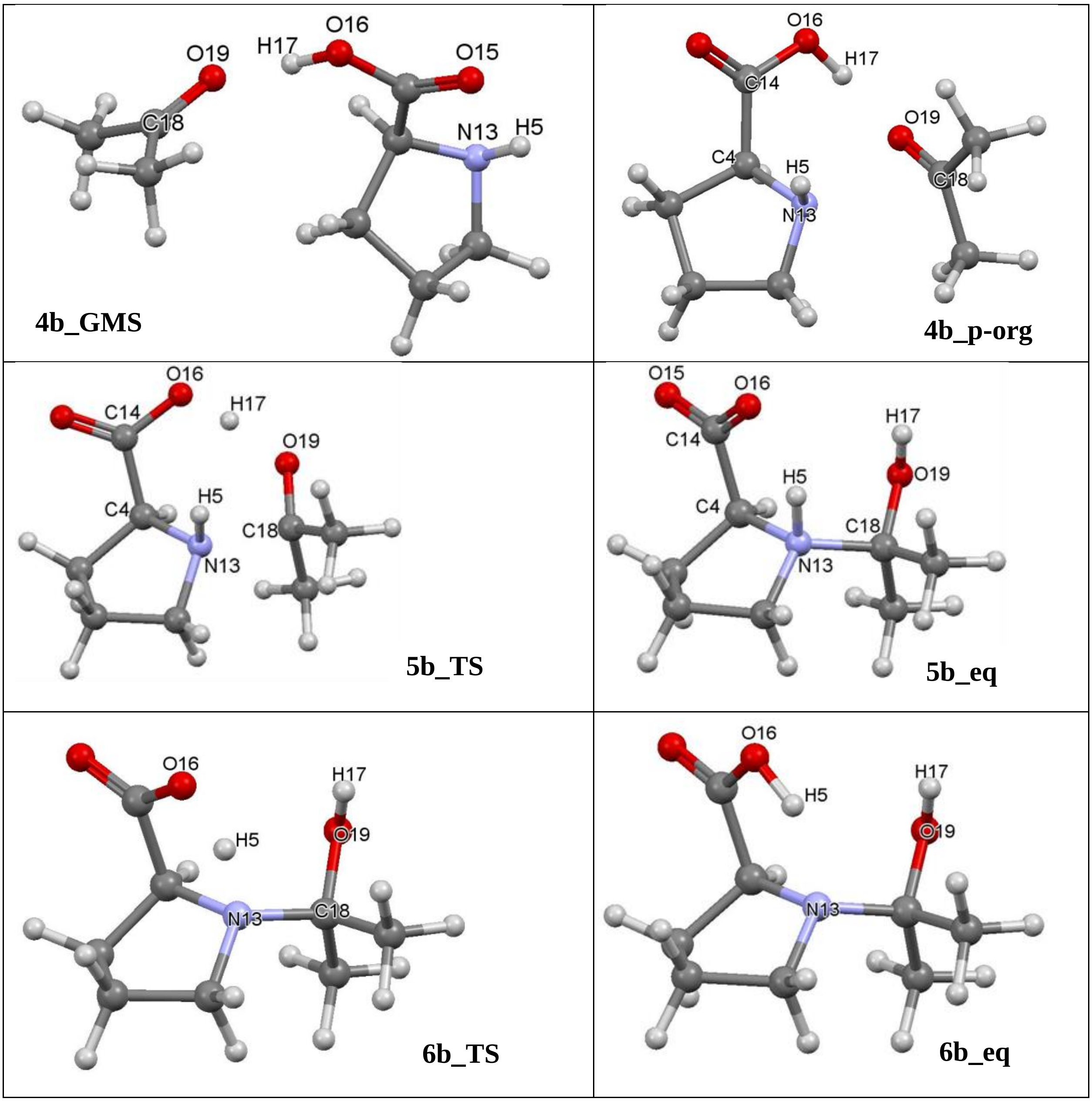

End of Part 2

Part 3

Search for global minimum and best pre-organized structures of 3-MCs

Data pertaining to 1a-containing 3-MCs

In general, the global minimum structure (GMS) of the 2-MC between proline **1** and acetone **2** must be most dominant in a solution and likely to be preferentially solvated. The DMSO molecule was placed such that its oxygen atom (O37) interacted with H5 of **1a** and the resultant structure was optimised in Gaussian – see **4A_inp** in Figure S1.

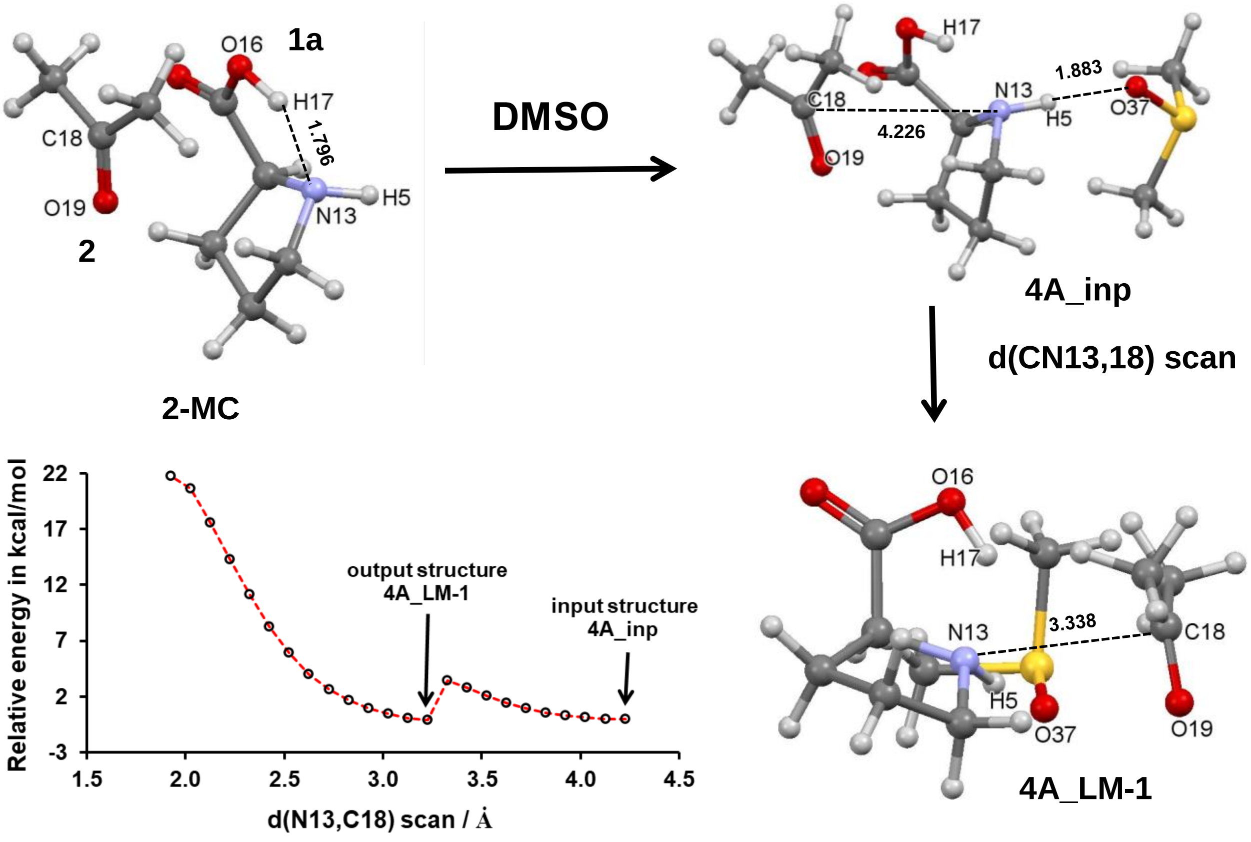

Figure S1. Structure of **2_MC,** made of proline **1a** and acetone **2,** used to generate **4A_inp** and data from the d(N13,C18) scan using **4A_inp** as an input structure that changed to the local minimum structure **4A_LM-1**.

Notably, there was no significant change in the structure of **4A_inp** before and after energy optimization. The d(N13,C18) reaction coordinate of **4A_inp** was then decreased to construct a bond between N13 of **1a** and C18 of **2**. This resulted in a local minimum structure **4A_LM-1** where, after energy optimization, the d(N13,C18) distance was 3.338 Å. Further decrease in d(N13,C18) distance resulted in a tremendous increase in the electronic energy of the molecular system due to the lack of proper pre-arrangement between the two molecules of proline (**1a**) and acetone (**2**).

Notably, the O16–H17···O19 classical hydrogen bond, which drives the formation of a formal bond between N13 of **1a** and C18 of **2**, is absent in **4A_LM-1**. To form the desired O16–H17···O19 hydrogen bond, either proline **1a** or acetone **2** must rotate, hence dihedral angle DA(H17,N13,C18,O19) made of atoms of proline **1a** and acetone **2** was scanned in steps of 10º resulting in the rotation of the molecule of acetone **2**. The scan data revealed a local minimum structure **4A_LM-2**; the orientation of proline relative to acetone in **4A_LM-2** is suitable and pre-organized for the N13–C18 bond formation - Figure S2.

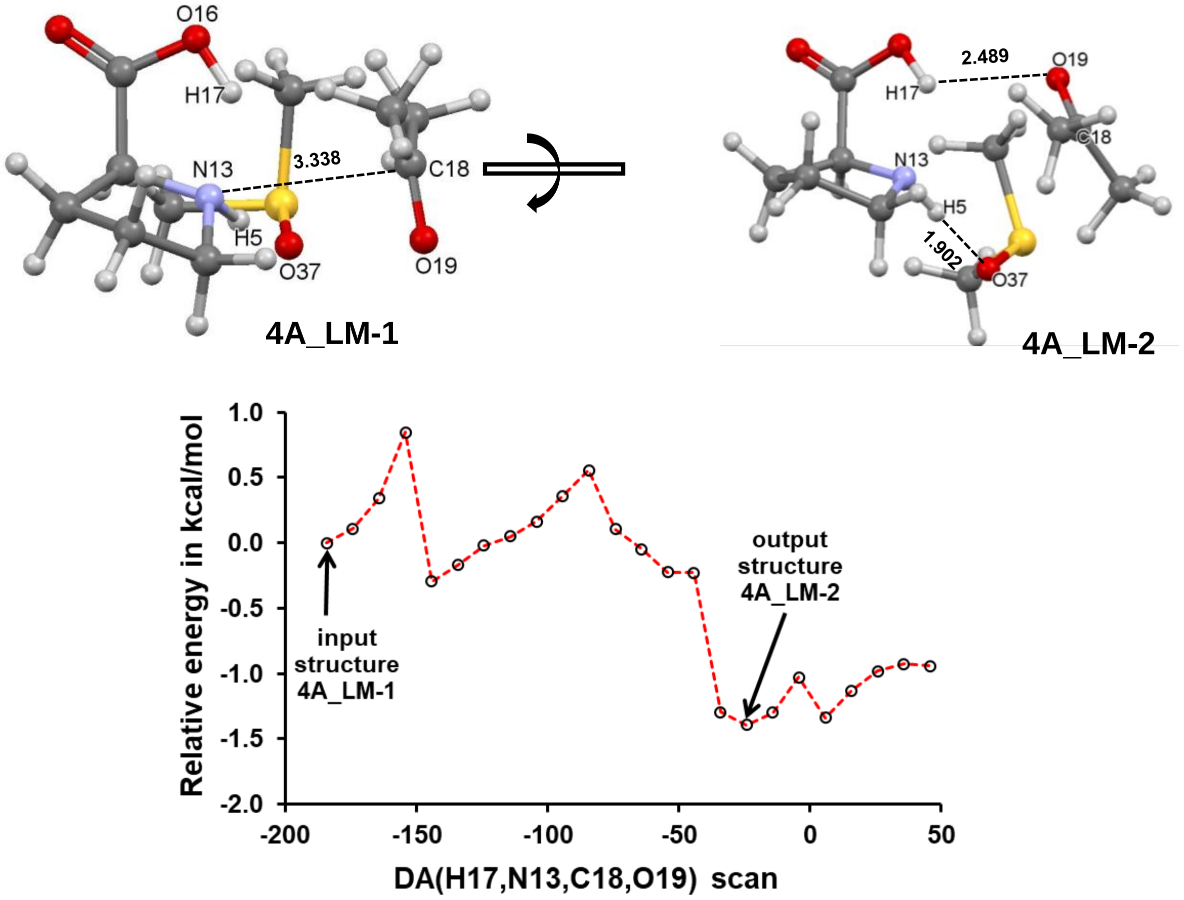

Figure S2. Data for the scan of DA(N13,H17,C18,O19) to construct the O16–H17···O19 hydrogen bond (required for the N13–C18 bond formation) leading to the rotation of acetone (**2**) and formation of **4A_LM-2**.

The analysis of net atomic charges revealed that H17 carries a significantly larger positive charge than H5 (both in proline **1**). Having such knowledge, most of orthodox organic chemists would expect that DMSO should in fact preferentially interact with H17. It was then of paramount importance to computationally establish the preferred mode of interaction between proline **1** and DMSO **3**. To achieve that, we placed DMSO **3** with O37 in close proximity to H17 in **1a** as a test – Figure S3. At the same time O19 of acetone **2** was facing H17 as this is required for the H17 transfer to acetone **2**. This means that the prepared structure **4A_inp*** allowed for a 'free' competition for the H17 between two H-bond type interactions involving O37 of DMSO **3** and

O19 of acetone **2**. The **4A_inp*** structure was optimised; the energy-optimized geometry **4A_LM-3** shows that the molecule of DMSO moved from top to bottom during energy optimization, indicating that the N13–H5···O37 H-bond is preferred over the O16–H17···O37 H-bond. (Figure S3).

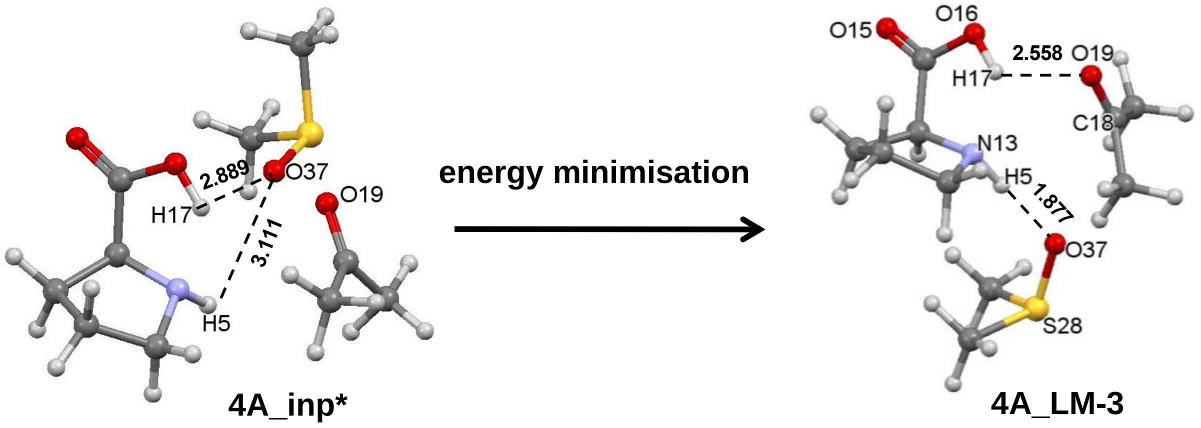

Figure S3. Ball and stick models for input structure **4A-inp*** and the resulting energy-optimized structure **4A_LM-3** used to establish the interaction mode of the DMSO solvent molecule.

In the search for the GMS for a 3-MC of proline **1a**, acetone **2** and the DMSO molecule **3**, **4A_LM-3** was submitted for the DA(N13,C1,C18,C20) scan. The resulting data (Figure S4) shows the lowest energy structure **4A_GMS**, which after energy optimization has the same free energy *G* as **4A_LM-3** but is marginally lower in E_{ZPVE} and *H* by ~ 1 kcal/mol.

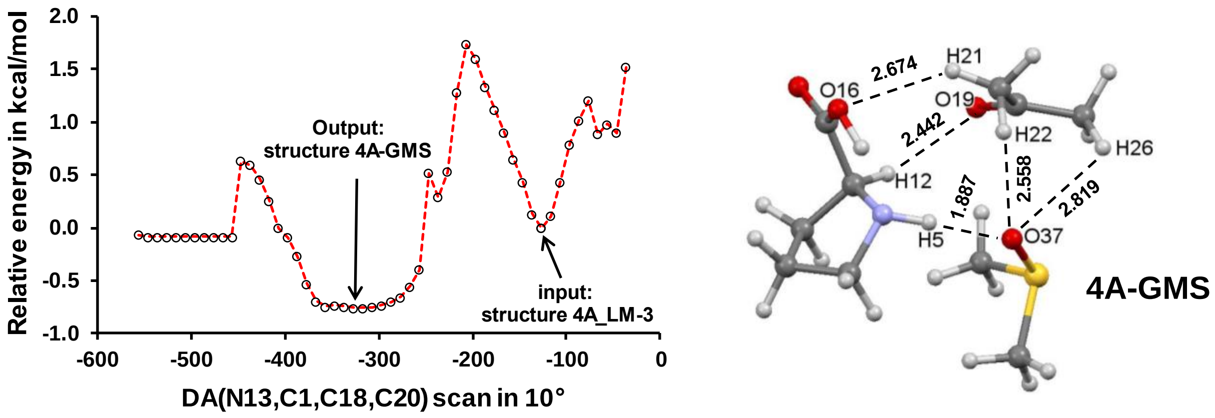

Figure S4. The DA(N13,C1,C18,C20) scan data obtained using **3A-LM3** as input in the search for the **4A-GMS**.

Data pertaining to 1b-containing 3-MCs

An initial protocol used to obtain a 3-MC of the HEC (**1b**), acetone (**2**), and DMSO solvent molecule (**3**) was the same as in the case of the LEC (**1a**). Unlike in **1a**-containing 2-MC which lacks the O16–H17···O19 hydrogen bond, **1b**-containing 2-MC has this classical hydrogen bond with d(H17,O19) of 1.684 Å (Figure S5). The DMSO solvent molecule was placed such that it interacted via the N13–H5···O37 hydrogen bond; after energy optimization, the resulting complex was named **4B_inp** (Figure S5). To construct a covalent bond between N13 of **1b** and C18 of **2**, the d(N13,C18) reaction coordinate of **4B-inp** was decreased resulting in two local minimum structures **4B_LM-1** and **4B_LM-2**.

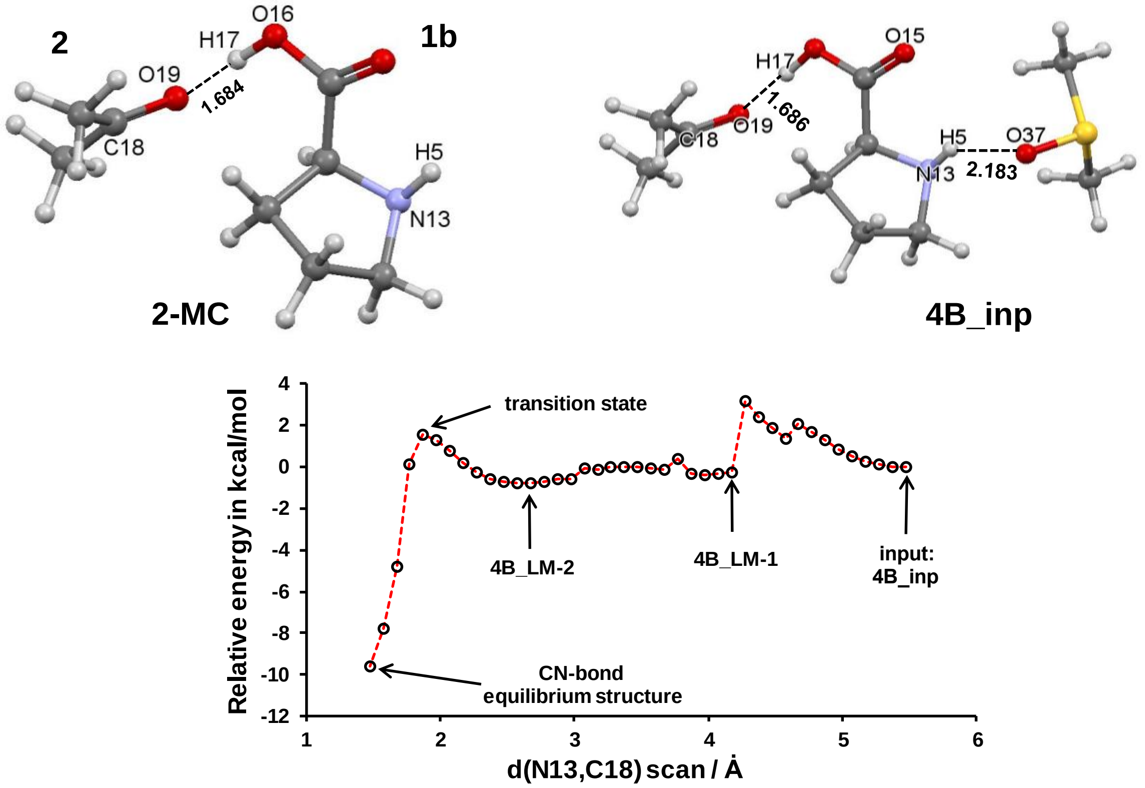

Figure S5. Ball and stick representation of the 2-MC of proline **1b** and acetone **2** and the **4B_inp** structure used as an input for the d(N13,C18) reaction coordinate scan from which two local minima **4B_LM-1** and **4B_LM-2** were found.

In the search of a global minimum structure, the energy-optimized complex (**4B_LM-2**) was submitted for DA(N13,C4,C20,C18) and DA(N13,H17,C18,O19) reaction coordinate scans. This resulted in the local minimum structure **4B_LM-3** and the lowest energy structure **4B_GMS**, the energy-optimized structures of all 4B complexes are shown in Figure 6 in the main body. Data obtained from the two scans is shown in Figure S6.

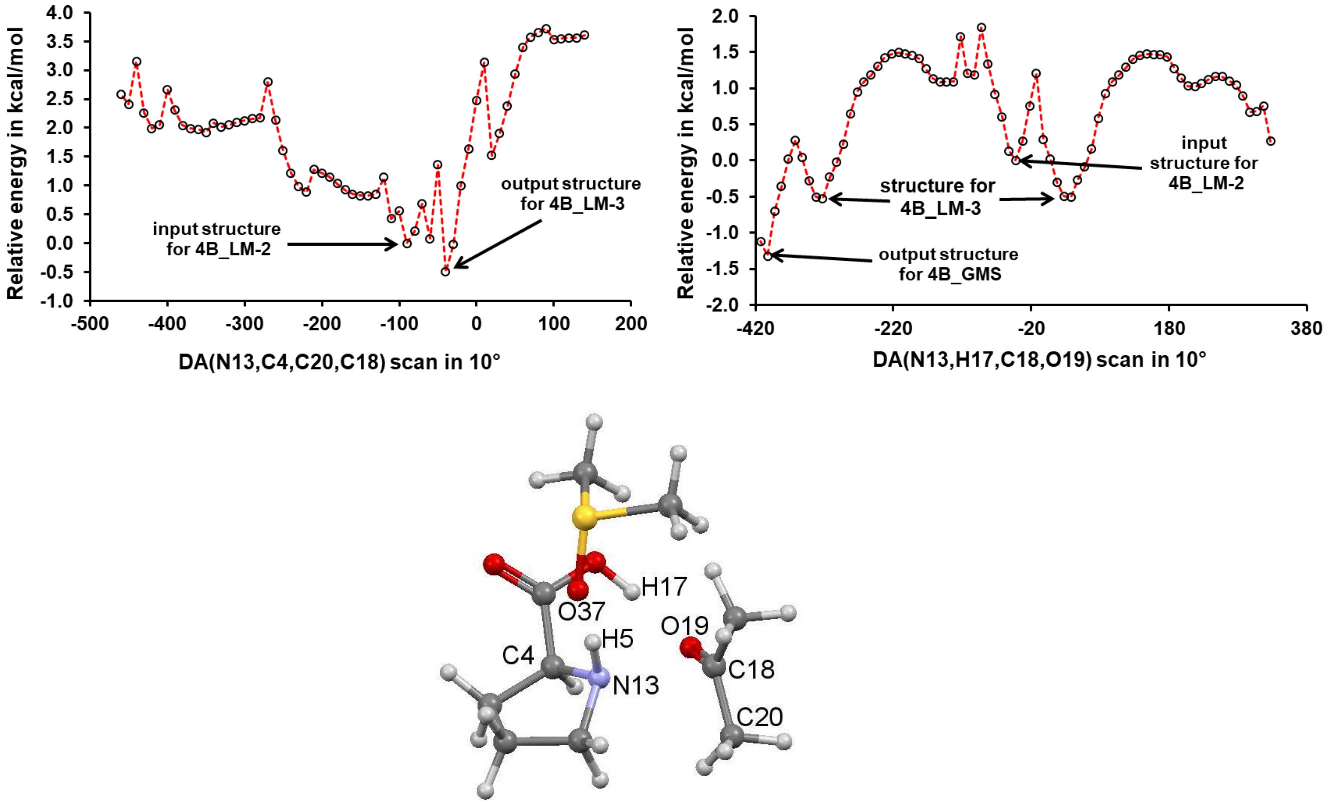

4B_LM-2

Figure S6. Ball and stick representation of **4B_LM-2** showing atoms selected in scanning dihedral angles DA(N13,C4,C20,C18) and DA(N13,H17,C18,O19) and the associated data from which **4B_LM-3** and **4B_GMS** were found.

Set of energies computed for all 3-MCs

Table S4. Energies (in a.u.) and, relative to reactants, differences in energies (in kcal/mol) for 3-MC made of proline (**1**), acetone (**2**) and an explicit DMSO solvent molecule (**3**). Data obtained at the B3LYP/6-311++G(d,p)/GD3 level in DMSO.

Part A – LEC-containing 3-MCs

	E	ΔE	E_{ZPVE}	ΔE_{ZPVE}	H	ΔH	G	ΔG
	3-MC							
**1a**+**2**+**3**	-1147.8284	0.0	-1147.5219	0.0	-1147.501	0.0	-1147.6103	0.0
4A_inp-1	-1147.8459	-11.0	-1147.5367	-9.3	-1147.5139	-8.1	-1147.5927	11.0
4A_LM-1	-1147.8463	-11.2	-1147.5370	-9.5	-1147.5143	-8.4	-1147.5925	11.1
4A_LM-2	-1147.8485	-12.6	-1147.5388	-10.6	-1147.5164	-9.7	-1147.5925	11.1
4A_inp-2	-1147.8252	2.0	-1147.5172	3.0	-1147.4967	2.7	-1147.5709	24.7
4A_LM-3	-1147.8491	-13.0	-1147.5395	-11.0	-1147.5170	-10.0	-1147.5942	10.1
4A_GMS	-1147.8503	-13.8	-1147.5407	-11.8	-1147.5183	-10.9	-1147.5943	10.0

Part B – HEC-containing 3-MCs

	E	ΔE	E_{ZPVE}	ΔE_{ZPVE}	H	ΔH	G	ΔG
	3-MC							
**1b**+**2**+**3**	-1147.8178	0.0	-1147.5113	0.0	-1147.4900	0.0	-1147.6001	0.0
4B_inp	-1147.8408	-14.5	-1147.5314	-12.6	-1147.5086	-11.7	-1147.5872	8.1
4B_LM-1	-1147.8414	-14.8	-1147.5317	-12.8	-1147.5090	-12.0	-1147.5865	8.5
4B_LM-2	-1147.8421	-15.3	-1147.5317	-12.8	-1147.5097	-12.3	-1147.5833	10.5
4B_LM-3	-1147.8429	-15.8	-1147.5334	-13.9	-1147.5108	-13.1	-1147.5870	8.2
4B_GMS	-1147.8442	-16.6	-1147.5338	-14.1	-1147.5118	-13.7	-1147.5862	8.8

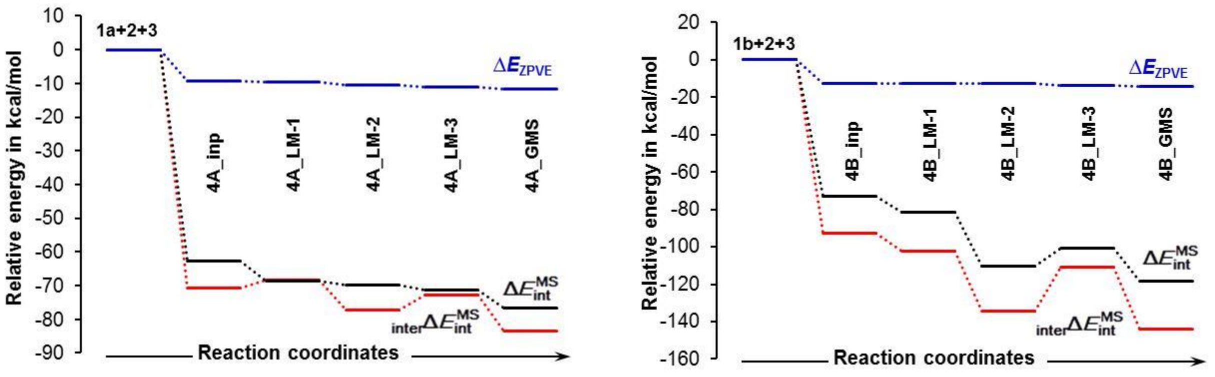

Figure S7. Relative to the energy of isolated molecules **1** (proline, either **1a** or **1b**), **2** (acetone) and **3** (DMSO solvent molecule), energy changes ΔE_{ZPVE}, $_{inter}\Delta E_{int}^{MS}$ and ΔE_{int}^{MS} computed for the indicated 3-MCs.

End of Part 3

Part 4

Largest net atomic charges in 3-MCs

Table S5. Net atomic charges (*Q*(A) in *e*) for: Part A - **4A** 3-MCs involving **1a** (LEC of proline), **2** (acetone) and **3** (DMSO) and Part B – **4B** 3-MCs involving **1b** (HEC of proline), **2** and **3**.

Part A: 4A 3-MCs

Inp-1		LM-1		LM-2		LM-3		GMS	
Atom	*Q*(A)	Atom	*Q*(A)	Atom	*Q*(A)	Atom	*Q*(A)	Atom	*Q*(A)
Atoms with largest negative net charges									
O37	-1.244	O37	-1.242	O37	-1.243	O37	-1.244	O37	-1.240
O15	-1.196	O15	-1.197	O15	-1.198	O15	-1.199	O15	-1.199
O19	-1.158	O19	-1.157	O19	-1.158	O19	-1.161	O19	-1.164
O16	-1.143	O16	-1.143	O16	-1.143	O16	-1.144	O16	-1.145
N13	-1.027	N13	-1.033	N13	-1.025	N13	-1.032	N13	-1.030
C29	-0.123	C33	-0.123	C33	-0.136	C29	-0.124	C33	-0.132
Atoms with largest positive net charges									
C1	0.320	C1	0.327	C1	0.328	C1	0.325	C1	0.322
H5	0.428	H5	0.431	H5	0.424	H5	0.427	H5	0.429
H17	0.602	H17	0.590	H17	0.609	H17	0.605	H17	0.599
C18	0.967	C18	0.967	C18	0.967	C18	0.963	C18	0.973
S28	1.133	S28	1.131	S28	1.122	S28	1.132	S28	1.126
C14	1.522	C14	1.522	C14	1.519	C14	1.522	C14	1.521

Part B: 4B 3MCs

Inp-1		LM-1		LM-2		LM-3		GMS	
Atom	*Q*(A)	Atom	*Q*(A)	Atom	*Q*(A)	Atom	*Q*(A)	Atom	*Q*(A)
Atoms with largest negative net charges									
O37	-1.243	O37	-1.240	O37	-1.247	O37	-1.244	O37	-1.247
O15	-1.183	O15	-1.180	O15	-1.179	O15	-1.181	O15	-1.184
O16	-1.162	O16	-1.159	O19	-1.178	O19	-1.172	O19	-1.178
O19	-1.151	O19	-1.157	O16	-1.157	O16	-1.153	O16	-1.158
N13	-0.982	N13	-0.981	N13	-0.967	N13	-0.984	N13	-0.969
C29	-0.135	C29	-0.129	C33	-0.128	C29	-0.135	C33	-0.133
Atoms with largest positive net charges									
C1	0.333	C1	0.325	C1	0.332	C1	0.331	C1	0.333
H5	0.380	H5	0.389	H5	0.407	H5	0.400	H5	0.409
H17	0.629	H17	0.633	H17	0.629	H17	0.629	H17	0.631
C18	0.940	C18	0.939	C18	0.955	C18	0.967	C18	0.953
S28	1.117	S28	1.118	S28	1.128	S28	1.117	S28	1.120
C14	1.523	C14	1.527	C14	1.522	C14	1.524	C14	1.524

Table S6. Atoms with the most negative and most positive charges in 3-MCs of LEC **4A** and HEC **4B**.

	**Atoms with most negative charge**					**Atoms with most positive charge**				
	**O37**	**O15**	**O19**	**O16**	**N13**	**C14**	**S28**	**C18**	**H17**	**H5**
	LEC									
4A_inp	-1.244	-1.196	-1.158	-1.143	-1.027	1.522	1.133	0.967	0.602	0.428
4A_LM-1	-1.242	-1.197	-1.157	-1.143	-1.033	1.522	1.131	0.967	0.590	0.431
4A_LM-2	-1.243	-1.198	-1.158	-1.143	-1.025	1.519	1.122	0.967	0.609	0.424
4A_LM-3	-1.244	-1.199	-1.161	-1.144	-1.032	1.522	1.132	0.963	0.605	0.427
4A_GMS	-1.240	-1.199	-1.164	-1.145	-1.030	1.521	1.126	0.973	0.599	0.429
Avr:	-1.243	-1.198	-1.160	-1.144	-1.029	1.521	1.129	0.967	0.601	0.428
StDev:	0.002	0.001	0.003	0.001	0.003	0.001	0.005	0.004	0.007	0.003
	HEC									
4B_inp	-1.243	-1.183	-1.151	-1.162	-0.982	1.523	1.117	0.940	0.629	0.380
4B_LM-1	-1.240	-1.180	-1.157	-1.159	-0.981	1.527	1.118	0.939	0.633	0.389
4B_LM-2	-1.247	-1.179	-1.178	-1.157	-0.967	1.522	1.128	0.955	0.629	0.407
4B_LM-3	-1.244	-1.181	-1.172	-1.153	-0.984	1.524	1.117	0.967	0.629	0.400
4B_GMS	-1.247	-1.184	-1.178	-1.158	-0.969	1.524	1.120	0.953	0.631	0.409
Avr:	-1.244	-1.181	-1.167	-1.158	-0.977	1.524	1.120	0.951	0.630	0.397
StDev:	0.003	0.002	0.012	0.003	0.008	0.002	0.005	0.012	0.002	0.012
	Difference: Average (HEC) minus Average (LEC)									
	-0.002	0.016	-0.008	-0.014	0.053	0.003	-0.009	-0.017	0.029	-0.031

End of Part 4

Part 5
Most significant intermolecular diatomic interactions in 3-MCs

Table S7. Top eight strongest attractive and repulsive diatomic intermolecular interactions (in kcal/mol) in the indicated **4A** 3-MCs involving **1a** (LEC of proline), **2** (acetone) and **3** (DMSO solvent molecule).

Part A. Molecules **1a** and **2** in **4A_inp-1**

Atom A of **1a**	Atom B of **2**	E_{int}(A,B)
Most attractive interactions		
C14	O19	-160.6
O16	C18	-113.4
O15	C18	-98.8
N13	C18	-80.2
H17	O19	-64.7
C1	O19	-34.7
H5	O19	-33.9
C4	O19	-29.9
Most repulsive interactions		
C4	C18	24.4
C1	C18	26.7
H5	C18	27.8
H17	C18	56.5
N13	O19	97.6
O15	O19	115.4
O16	O19	124.7
C14	C18	137.9

Part B. Molecules **1a** and **3** in **4A_inp-1**

Atom A of **1a**	Atom B of **3**	E_{int}(A,B)
Most significant attractive		
C14	O37	-123.7
N13	S28	-109.2
H5	O37	-94.4
O16	S28	-80.0
O15	S28	-76.1
H17	O37	-58.1
C1	O37	-38.9
C4	O37	-36.1
Most significant repulsive		
C4	S28	29.3
C1	S28	31.0
H17	S28	46.8
H5	S28	59.6
O15	O37	86.6
O16	O37	96.6
C14	S28	105.8
N13	O37	134.0

Part C. Molecules **2** and **3** in **4A_inp-1**

Atom A of **2**	Atom B of **3**	E_{int}(A,B)
Most significant attractive		
C18	O37	-128.9
O19	S28	-121.7
O19	H34	-16.0
H22	O37	-11.6
C18	C33	-11.6
H26	O37	-7.4
O19	H35	-7.3
C18	C29	-7.3
Most significant repulsive		
C18	H36	5.0
C18	H35	5.8
H22	S28	6.2
O19	C29	8.6
C18	H34	8.8
O19	C33	12.9
C18	S28	105.0
O19	O37	140.2

Part D. Molecules **1a** and **2** in **4A_LM-1**

Atom A of **1a**	Atom B of **2**	E_{int}(A,B)
Most significant attractive		
C14	O19	-103.5
N13	C18	-102.7
O16	C18	-84.8
O15	C18	-61.6
H17	O19	-57.7
H5	O19	-48.7
C1	O19	-38.1
C4	O19	-26.8
Most significant repulsive		
C4	C18	23.3
C1	C18	31.1
H5	C18	41.6
H17	C18	53.5
O15	O19	69.6
O16	O19	92.0
C14	C18	92.7
N13	O19	114.9

Part E. Molecules **1a** and **3** in **4A_LM-1**

Atom A of **1a**	Atom B of **3**	E_{int}(A,B)
Most significant attractive		
C14	O37	-134.9
N13	S28	-111.3
O16	S28	-96.9
H5	O37	-94.2
O15	S28	-87.8
H17	O37	-63.0
C1	O37	-38.1
C4	O37	-36.8
Most significant repulsive		
C1	S28	30.0
C4	S28	30.3
H17	S28	54.4
H5	S28	60.0
O15	O37	93.7
O16	O37	107.6
C14	S28	123.5
N13	O37	135.2

Part F. Molecules **2** and **3** in **4A_LM-1**

Atom A of **2**	Atom B of **3**	E_{int}(A,B)
Most significant attractive		
C18	O37	-96.0
O19	S28	-90.2
H21	O37	-14.2
C18	C33	-7.6
C18	C29	-6.0
H23	O37	-5.4
O19	H35	-4.6
O19	H34	-4.6
Most significant repulsive		
C18	H34	4.0
C18	H35	4.1
H23	S28	4.5
H21	S28	7.1
O19	C29	7.1
O19	C33	8.6
C18	S28	76.7
O19	O37	114.2

Part G. Molecules **1a** and **2** in **4A_LM-2**

Atom A of **1a**	Atom B of **2**	E_{int}(A,B)
Most significant attractive		
C14	O19	-136.8
N13	C18	-102.8
O16	C18	-102.5
H17	O19	-90.8
O15	C18	-69.4
H5	O19	-48.9
C1	O19	-32.3
C4	O19	-30.2
Most significant repulsive		
C4	C18	24.7
C1	C18	27.6
H5	C18	41.5
H17	C18	67.7
O15	O19	88.7
C14	C18	106.1
N13	O19	119.5
O16	O19	132.5

Part H. Molecules **1a** and **3** in **4A_LM-2**

Atom A of **1a**	Atom B of **3**	E_{int}(A,B)
Most significant attractive		
C14	O37	-131.8
N13	S28	-111.6
O16	S28	-94.1
H5	O37	-93.4
O15	S28	-85.5
H17	O37	-62.1
C1	O37	-38.4
C4	O37	-37.8
Most significant repulsive		
C1	S28	30.1
C4	S28	30.9
H17	S28	54.8
H5	S28	59.7
O15	O37	92.5
O16	O37	103.2
C14	S28	120.2
N13	O37	134.8

Part I. Molecules **2** and **3** in **4A_LM-2**

Atom A of **2**	Atom B of **3**	E_{int}(A,B)
Most significant attractive		
O19	C14	-136.8
C18	N13	-102.8
C18	O16	-102.5
O19	H17	-90.8
C18	O15	-69.4
O19	H5	-48.9
O19	C1	-32.3
O19	C4	-30.2
Most significant repulsive		
C18	C4	24.7
C18	C1	27.6
C18	H5	41.5
C18	H17	67.7
O19	O15	88.7
C18	C14	106.1
O19	N13	119.5
O19	O16	132.5

Part J. Molecules **1a** and **2** in **4A_LM-3**

Atom A of **1a**	Atom B of **2**	E_{int}(A,B)
Most significant attractive		
C14	O19	-132.6
N13	C18	-111.0
O16	C18	-104.9
H17	O19	-89.5
O15	C18	-69.8
H5	O19	-47.2
C1	O19	-36.6
C4	O19	-30.9
Most significant repulsive		
C4	C18	26.0
C1	C18	30.5
H5	C18	42.2
H17	C18	70.3
O15	O19	85.6
C14	C18	107.7
N13	O19	123.9
O16	O19	129.7

Part K. Molecules **1a** and **3** in **4A_LM-3**

Atom A of **1a**	Atom B of **3**	E_{int}(A,B)
Most significant attractive		
C14	O37	-123.9
N13	S28	-112.3
H5	O37	-95.8
O16	S28	-79.5
O15	S28	-75.2
H17	O37	-58.5
C1	O37	-39.7
C4	O37	-37.1
Most significant repulsive		
C4	S28	30.4
C1	S28	32.5
H17	S28	46.9
H5	S28	60.9
O15	O37	86.9
O16	O37	97.0
C14	S28	104.7
N13	O37	136.1

Part L. Molecules **2** and **3** in **4A_LM-3**

Atom A of **2**	Atom B of **3**	E_{int}(A,B)
Most significant attractive		
C18	O37	-94.8
O19	S28	-80.1
H22	O37	-15.6
H26	O37	-13.7
C18	C29	-6.6
C18	C33	-6.0
H23	O37	-4.6
H25	O37	-4.6
Most significant repulsive		
C18	H32	3.6
C18	H31	3.6
H26	S28	6.4
O19	C33	6.8
H22	S28	7.3
O19	C29	7.4
C18	S28	73.2
O19	O37	101.7

Part M. Molecules **1a** and **2** in **4A_GMS**

Atom A of **1a**	Atom B of **2**	E_{int}(A,B)
Most significant attractive		
C14	O19	-153.5
O15	C18	-89.3
O16	C18	-88.1
N13	C18	-85.0
H17	O19	-55.9
H5	O19	-47.2
C4	O19	-32.9
C1	O19	-26.8
Most significant repulsive		
C1	C18	22.1
C4	C18	25.1
H5	C18	39.9
H17	C18	46.8
N13	O19	101.8
O16	O19	106.9
O15	O19	115.7
C14	C18	120.3

Part N. Molecules **1a** and **3** in **4A_GMS**

Atom A of **1a**	Atom B of **3**	E_{int}(A,B)
Most significant attractive		
C14	O37	-123.3
N13	S28	-111.6
H5	O37	-95.5
O16	S28	-79.0
O15	S28	-74.9
H17	O37	-57.7
C1	O37	-39.3
C4	O37	-35.3
Most significant repulsive		
C4	S28	28.9
C1	S28	32.3
H17	S28	46.3
H5	S28	60.9
O15	O37	86.6
O16	O37	96.3
C14	S28	104.2
N13	O37	134.9

Part O. Molecules **2** and **3** in **4A_GMS**

Atom A of **2**	Atom B of **3**	E_{int}(A,B)
Most significant attractive		
C18	O37	-128.9
O19	S28	-121.7
O19	H34	-16.0
H22	O37	-11.6
C18	C33	-11.6
H26	O37	-7.4
O19	H35	-7.3
C18	C29	-7.3
Most significant repulsive		
C18	H36	5.0
C18	H35	5.8
H22	S28	6.2
O19	C29	8.6
C18	H34	8.8
O19	C33	12.9
C18	S28	105.0
O19	O37	140.2

Table S8. Top eight strongest attractive and repulsive diatomic intermolecular interactions (in kcal/mol) in the indicated **4B** 3-MCs involving **1b** (HEC of proline), **2** (acetone) and **3** (DMSO solvent molecule).

Part A. Molecules **1b** and **2** in **4B_inp**

Atom A of **1b**	Atom B of **2**	E_{int}(A,B)
Most significant attractive		
C14	O19	-158.8
H17	O19	-143.5
O16	C18	-114.5
O15	C18	-73.7
N13	C18	-58.1
C4	O19	-31.2
H5	O19	-27.6
C1	O19	-25.7
Most significant repulsive		
C1	C18	19.8
H5	C18	21.1
C4	C18	22.4
N13	O19	76.7
H17	C18	85.6
O15	O19	97.5
C14	C18	117.1
O16	O19	150.0

Part B. Molecules **1b** and **3** in **4B_inp**

Atom A of **1b**	Atom B of **3**	E_{int}(A,B)
Most significant attractive		
C14	O37	-141.1
N13	S28	-112.2
O15	S28	-110.8
O16	S28	-73.6
H5	O37	-73.1
C1	O37	-42.2
H17	O37	-41.5
C4	O37	-35.4
Most significant repulsive		
C4	S28	30.3
C1	S28	34.8
H17	S28	36.6
H5	S28	53.5
O16	O37	83.5
C14	S28	123.0
N13	O37	124.8
O15	O37	126.4

Part C. Molecules **2** and **3** in **4B_inp**

Atom A of **2**	Atom B of **3**	E_{int}(A,B)
Most significant attractive		
O19	S28	-55.2
C18	O37	-48.7
C18	C29	-5.0
C18	C33	-4.9
O19	H36	-4.2
O19	H32	-4.2
O19	H34	-2.9
O19	H35	-2.9
Most significant repulsive		
C18	H34	2.2
H21	S28	2.4
C18	H32	3.2
C18	H36	3.2
O19	C33	6.3
O19	C29	6.5
C18	S28	42.9
O19	O37	62.4

Part D. Molecules **1b** and **2** in **4B_LM-1**

Atom A of **1b**	Atom B of **2**	E_{int}(A,B)
Most significant attractive		
C14	O19	-157.8
H17	O19	-142.1
O16	C18	-111.6
N13	C18	-81.2
O15	C18	-72.4
C4	O19	-35.2
H5	O19	-33.8
C1	O19	-29.7
Most significant repulsive		
C1	C18	24.0
C4	C18	26.3
H5	C18	26.9
H17	C18	83.9
O15	O19	96.6
N13	O19	101.0
C14	C18	115.5
O16	O19	149.0

Part E. Molecules **1b** and **3** in **4B_LM-1**

Atom A of **1b**	Atom B of **3**	E_{int}(A,B)
Most significant attractive		
C14	O37	-158.4
N13	S28	-117.1
O15	S28	-104.9
O16	S28	-97.2
H5	O37	-83.3
H17	O37	-53.4
C1	O37	-38.8
C4	O37	-36.8
Most significant repulsive		
C4	S28	31.4
C1	S28	33.0
H17	S28	50.0
H5	S28	57.5
O16	O37	105.6
O15	O37	125.3
N13	O37	128.8
C14	S28	136.5

Part F. Molecules **2** and **3** in **4B_LM-1**

Atom A of **2**	Atom B of **3**	E_{int}(A,B)
Most significant attractive		
O19	S28	-74.2
C18	O37	-62.3
C18	C33	-8.0
H25	O37	-7.7
C18	C29	-7.2
O19	H36	-6.1
O19	H32	-5.2
O19	H34	-5.0
Most significant repulsive		
C18	H34	4.0
C18	H32	4.3
C18	H36	4.8
H25	S28	7.5
O19	C29	8.6
O19	C33	9.8
C18	S28	60.2
O19	O37	77.5

Part G. Molecules **1b** and **2** in **4B_LM-2**

Atom A of **1b**	Atom B of **2**	E_{int}(A,B)
Most significant attractive		
C14	O19	-168.4
H17	O19	-148.4
N13	C18	-130.9
O16	C18	-122.5
O15	C18	-80.2
H5	O19	-46.9
C4	O19	-42.0
C1	O19	-37.2
Most significant repulsive		
C1	C18	32.3
C4	C18	32.9
H5	C18	40.9
H17	C18	91.4
O15	O19	102.6
C14	C18	129.2
N13	O19	129.2
O16	O19	156.5

Part H. Molecules **1b** and **3** in **4B_LM-2**

Atom A of **1b**	Atom B of **3**	E_{int}(A,B)
Most significant attractive		
C14	O37	-171.7
O15	S28	-123.2
O16	S28	-103.0
N13	S28	-100.8
H5	O37	-89.2
H17	O37	-61.1
C1	O37	-40.5
C4	O37	-36.8
Most significant repulsive		
C4	S28	29.7
C1	S28	31.5
H17	S28	50.9
H5	S28	55.3
O16	O37	120.1
N13	O37	126.2
O15	O37	130.9
C14	S28	152.1

Part I. Molecules **2** and **3** in **4B_LM-2**

Atom A of **2**	Atom B of **3**	E_{int}(A,B)
Most significant attractive		
O19	S28	-81.8
C18	O37	-80.5
C18	C33	-7.6
C18	C29	-6.9
O19	H34	-6.0
O19	H36	-4.9
O19	H35	-4.6
H26	O37	-4.4
Most significant repulsive		
C18	H35	3.7
H26	S28	3.9
C18	H36	4.0
C18	H34	4.9
O19	C29	8.0
O19	C33	9.3
C18	S28	69.7
O19	O37	93.6

Part J. Molecules **1b** and **2** in **4B_LM-3**

Atom A of **1b**	Atom B of **2**	E_{int}(A,B)
Most significant attractive		
C14	O19	-155.2
H17	O19	-135.4
N13	C18	-120.5
O16	C18	-116.8
O15	C18	-75.6
H5	O19	-47.8
C4	O19	-36.3
C1	O19	-34.9
Most significant repulsive		
C4	C18	30.3
C1	C18	30.6
H5	C18	39.9
H17	C18	87.7
O15	O19	96.0
C14	C18	120.5
N13	O19	129.0
O16	O19	150.0

Part K. Molecules **1b** and **3** in **4B_LM-3**

Atom A of **1b**	Atom B of **3**	E_{int}(A,B)
Most significant attractive		
C14	O37	-176.9
O16	S28	-117.1
O15	S28	-105.6
N13	S28	-105.3
H5	O37	-88.1
H17	O37	-69.7
C4	O37	-39.7
C1	O37	-39.6
Most significant repulsive		
C1	S28	30.8
C4	S28	31.2
H5	S28	55.9
H17	S28	62.9
O15	O37	126.3
N13	O37	127.6
O16	O37	130.5
C14	S28	147.8

Part L. Molecules **2** and **3** in **4B_LM-3**

Atom A of **2**	Atom B of **3**	E_{int}(A,B)
Most significant attractive		
O19	S28	-100.1
C18	O37	-84.6
O19	H32	-17.3
C18	C29	-11.1
C18	C33	-8.3
O19	H36	-7.6
O19	H30	-6.9
O19	H31	-6.7
Most significant repulsive		
C18	H31	5.1
C18	H30	5.2
C18	H36	5.7
C18	H32	8.8
O19	C33	11.0
O19	C29	12.8
C18	S28	78.2
O19	O37	106.1

Part M. Molecules **1b** and **2** in **4B_GMS**

Atom A of **1b**	Atom B of **2**	E_{int}(A,B)
Most significant attractive		
C14	O19	-169.4
H17	O19	-150.6
N13	C18	-130.4
O16	C18	-122.6
O15	C18	-80.5
H5	O19	-47.4
C4	O19	-42.1
C1	O19	-37.2
Most significant repulsive		
C1	C18	32.2
C4	C18	32.9
H5	C18	41.2
H17	C18	92.0
O15	O19	103.3
N13	O19	129.4
C14	C18	129.5
O16	O19	156.9

Part N. Molecules **1b** and **3** in **4B_GMS**

Atom A of **1b**	Atom B of **3**	E_{int}(A,B)
Most significant attractive		
C14	O37	-171.7
O15	S28	-123.2
O16	S28	-103.0
N13	S28	-100.8
H5	O37	-89.2
H17	O37	-61.1
C1	O37	-40.5
C4	O37	-36.8
Most significant repulsive		
C4	S28	29.7
C1	S28	31.5
H17	S28	50.9
H5	S28	55.3
O16	O37	120.1
N13	O37	126.2
O15	O37	130.9
C14	S28	152.1

Part O. Molecules **2** and **3** in **4B_GMS**

Atom A of **2**	Atom B of **3**	E_{int}(A,B)
Most significant attractive		
C18	O37	-82.2
O19	S28	-78.1
C18	C29	-6.9
C18	C33	-6.5
O19	H32	-5.8
O19	H36	-5.4
H21	O37	-4.4
H23	O37	-4.2
Most significant repulsive		
C18	H30	3.1
H21	S28	3.3
C18	H36	4.4
C18	H32	4.7
O19	C33	7.9
O19	C29	8.4
C18	S28	65.6
O19	O37	96.0

End of Part 5

Part 6

Inter-fragment and selected atom-fragment interaction energies obtained for $\mathcal{A}$, $\mathcal{P}$n and $\mathcal{D}$ in 3-MCs

Part A

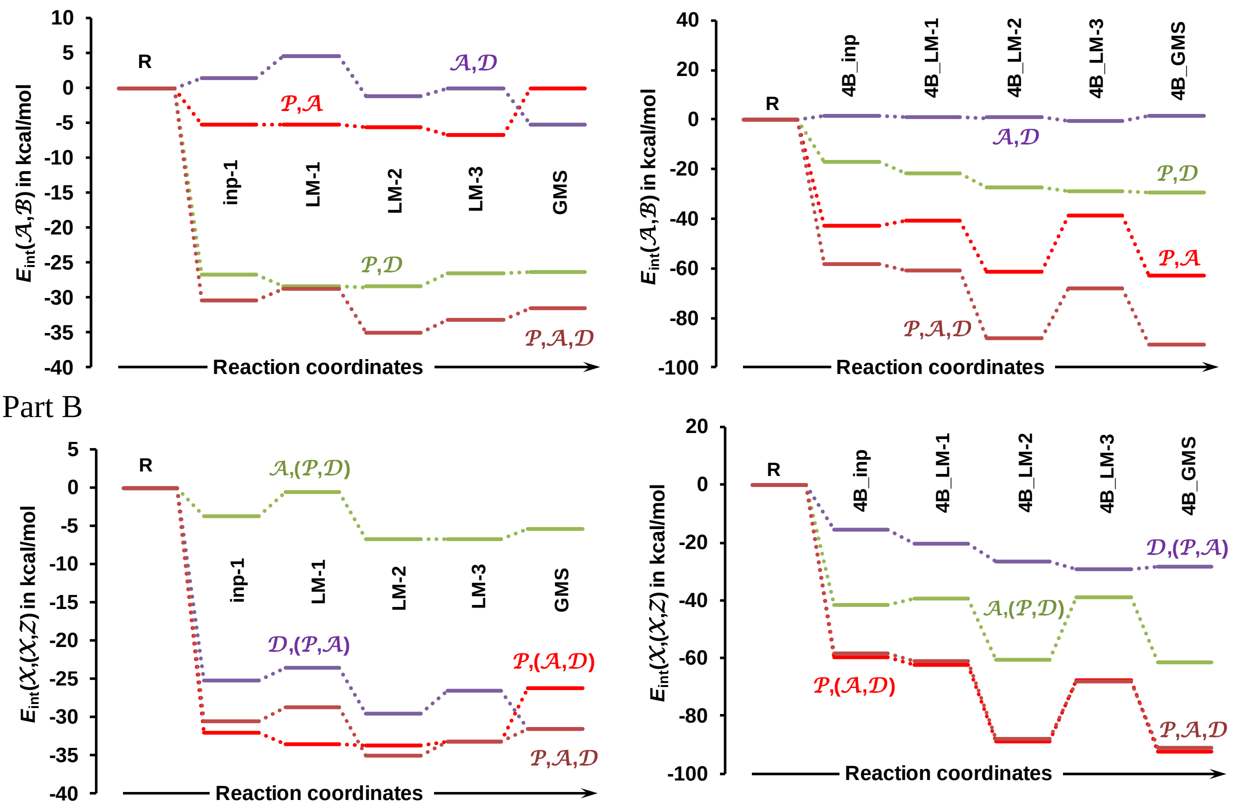

Figure S8. Interaction between atomic fragments {$\mathcal{P}$,$\mathcal{A}$}, {$\mathcal{P}$,$\mathcal{D}$}, {$\mathcal{A}$,$\mathcal{D}$} and {$\mathcal{P}$,$\mathcal{A}$,$\mathcal{D}$} in Part A and an atomic fragment and two remaining atomic fragments in Part B $\mathcal{A}$,($\mathcal{P}$,$\mathcal{D}$), $\mathcal{D}$($\mathcal{P}$,$\mathcal{A}$), $\mathcal{P}$($\mathcal{A}$,$\mathcal{D}$) and $\mathcal{P}$,$\mathcal{A}$,$\mathcal{D}$ in the 3-MCs considered in this work.

Part A

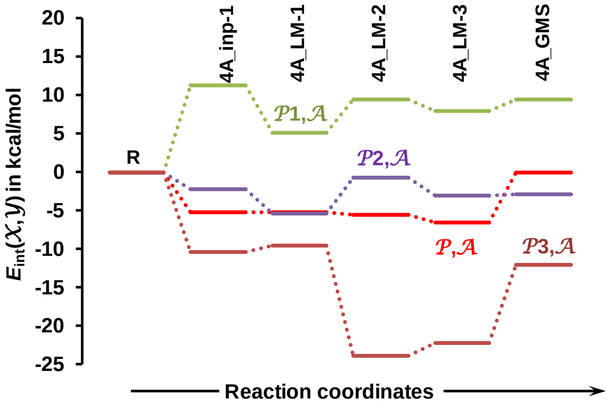

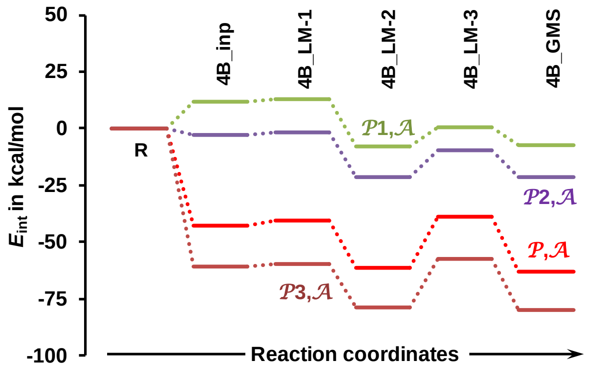

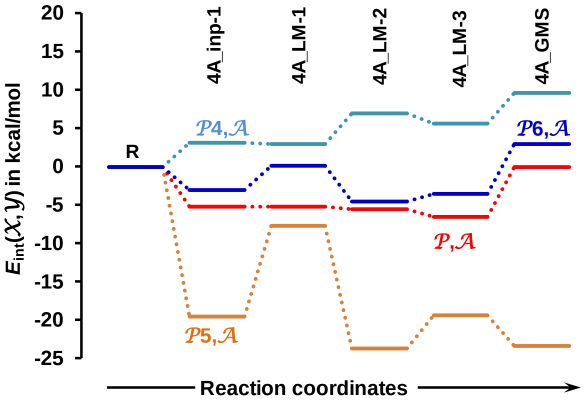

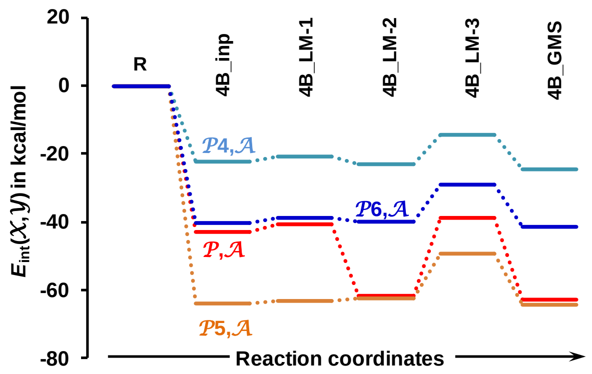

Part B

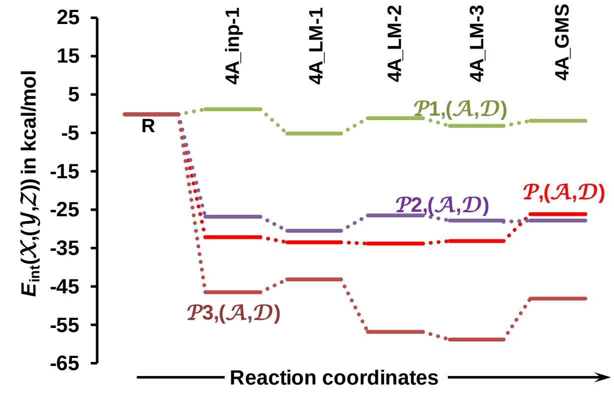

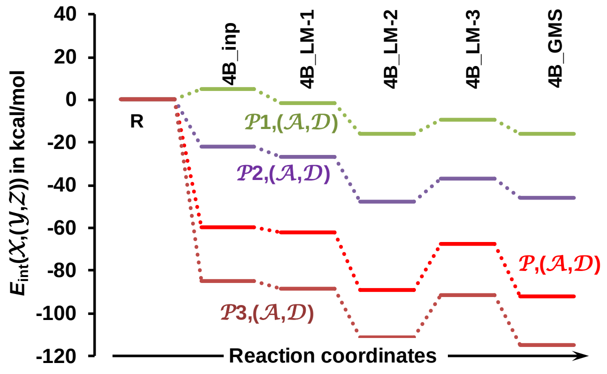

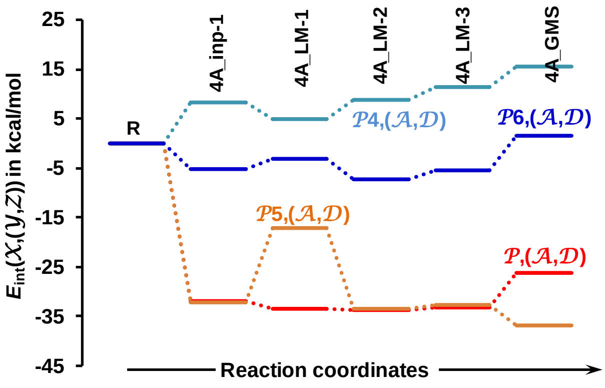

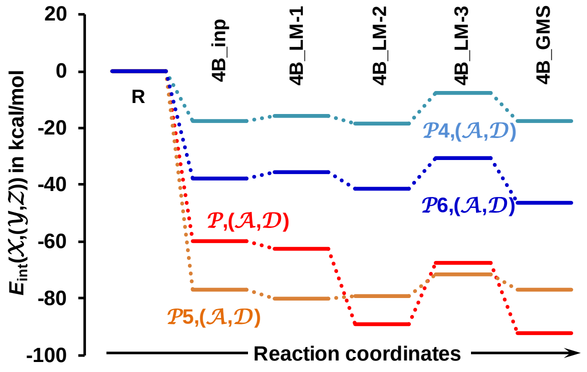

Figure S9. Interaction between the 𝒫n fragment (𝒫, 𝒫1, 𝒫2, 𝒫3, 𝒫4, 𝒫5 and 𝒫6) made of atoms of proline and fragment 𝒜 of acetone in Part A and interaction between the same fragment of proline with the combined fragments made of atoms 𝒜 of acetone and 𝒟 of DMSO (𝒜,𝒟) in Part B.

Table S9. Strongest inter-molecular di-atomic interaction energies (in kcal/mol) between selected atoms of **1** and atoms (C18,O19) of **2** and atoms (S28,O37) of **3** in the indicated 3-MC involving **1** (proline), **2** (acetone) and **3** (DMSO solvent molecule).

Part A LEC

	4A_LM-3				4A_GMS			
Atom	C18	O19	S28	O37	C18	O19	S28	O37
C1	30.5	-36.6	32.5	-39.7	22.1	-26.8	32.3	-39.3
C4	26.0	-30.9	30.4	-37.1	25.1	-32.9	28.9	-35.3
H5	42.2	-47.2	60.9	-95.8	39.9	-47.2	60.9	-95.5
N13	-111.0	123.9	-112.3	136.1	-85.0	101.8	-111.6	134.9
C14	107.7	-132.6	104.7	-123.9	120.3	-153.5	104.2	-123.3
O15	-69.8	85.6	-75.2	86.9	-89.3	115.7	-74.9	86.6
O16	-104.9	129.7	-79.5	97.0	-88.1	106.9	-79.0	96.3
H17	70.3	-89.5	46.9	-58.5	46.8	-55.9	46.3	-57.7

Part B HEC

	4B_LM-3				4B_GMS			
Atom	C18	O19	S28	O37	C18	O19	S28	O37
C1	30.6	-34.9	30.8	-39.6	32.2	-37.2	31.5	-40.5
C4	30.3	-36.3	31.2	-39.7	32.9	-42.1	29.7	-36.8
H5	39.9	-47.8	55.9	-88.1	41.2	-47.4	55.3	-89.2
N13	-120.5	129	127.6	-105.3	-130.4	129.4	126.2	-100.8
C14	120.5	-155.2	147.8	-176.9	129.5	-169.4	152.1	-171.7
O15	-75.6	96	126.3	-105.6	-80.5	103.3	130.9	-123.2
O16	-116.8	150	130.5	-117.1	-122.6	156.9	120.1	-103
H17	87.7	-135.4	62.9	-69.7	92	-150.6	50.9	-61.1

Table S10. Strongest inter-molecular di-atomic interaction energies (in kcal/mol) between atoms of **2** (C18,O19) and atoms of either **1** or **3** (S28,O37) in the indicated 3-MC involving **1** (proline), **2** (acetone) and **3** (DMSO solvent molecule).

Part A LEC

Atom	C1	C4	H5	N13	C14	O15	O16	H17	S28	O37
	4A_LM-3									
C18	30.5	26.0	42.2	-111.0	107.7	-69.8	-104.9	70.3	73.2	-94.8
O19	-36.6	-30.9	-47.2	123.9	-132.6	85.6	129.7	-89.5	-80.1	101.7
	4A_GMS									
C18	22.1	25.1	39.9	-85.0	120.3	-89.3	-88.1	46.8	105.0	-128.9
O19	-26.8	-32.9	-47.2	101.8	-153.5	115.7	106.9	-55.9	-121.7	140.2

Part B HEC

Atom	C1	C4	H5	N13	C14	O15	O16	H17	S28	O37
	4B_LM-3									
C18	30.6	30.3	39.9	-120.5	120.5	-75.6	-116.8	87.7	78.2	-84.6
O19	-34.9	-36.3	-47.8	129	-155.2	96	150	-135.4	-100.1	106.1
	4B_GMS									
C18	32.2	32.9	41.2	-130.4	129.5	-80.5	-122.6	92	65.6	-82.2
O19	-37.2	-42.1	-47.4	129.4	-169.4	103.3	156.9	-150.6	-78.1	96

Table S11. Strongest inter-molecular di-atomic interaction energies (in kcal/mol) between atoms of **3** (S28,O37) and atoms of either **1** (C1,C4, H5, N13, C14, O15, O16, H17) or **2** (C18,O19) in the indicated 3-MCs for both LEC Part A and HEC Part B.

Part A LEC

**Atom**	**C1**	**C4**	**H5**	**N13**	**C14**	**O15**	**O16**	**H17**	**C18**	**O19**
	**4A_LM-3**									
S28	32.5	30.4	60.9	-112.3	104.7	-75.2	-79.5	46.9	73.2	-80.1
O37	-39.7	-37.1	-95.8	136.1	-123.9	86.9	97.0	-58.5	-94.8	101.7
	**4A_GMS**									
S28	32.3	28.9	60.9	-111.6	104.2	-74.9	-79.0	46.3	105.0	-121.7
O37	-39.3	-35.3	-95.5	134.9	-123.3	86.6	96.3	-57.7	-128.9	140.2

Part B HEC

**Atom**	**C1**	**C4**	**H5**	**N13**	**C14**	**O15**	**O16**	**H17**	**C18**	**O19**
	**4B_LM-3**									
S28	30.8	31.2	55.9	-105.3	147.8	-105.6	-117.1	62.9	78.2	-100.1
O37	-39.6	-39.7	-88.1	127.6	-176.9	126.3	130.5	-69.7	-84.6	106.1
	**4B_GMS**									
S28	31.5	29.7	55.3	-100.8	152.1	-123.2	-103	50.9	65.6	-78.1
O37	-40.5	-36.8	-89.2	126.2	-171.7	130.9	120.1	-61.1	-82.2	96

Part A

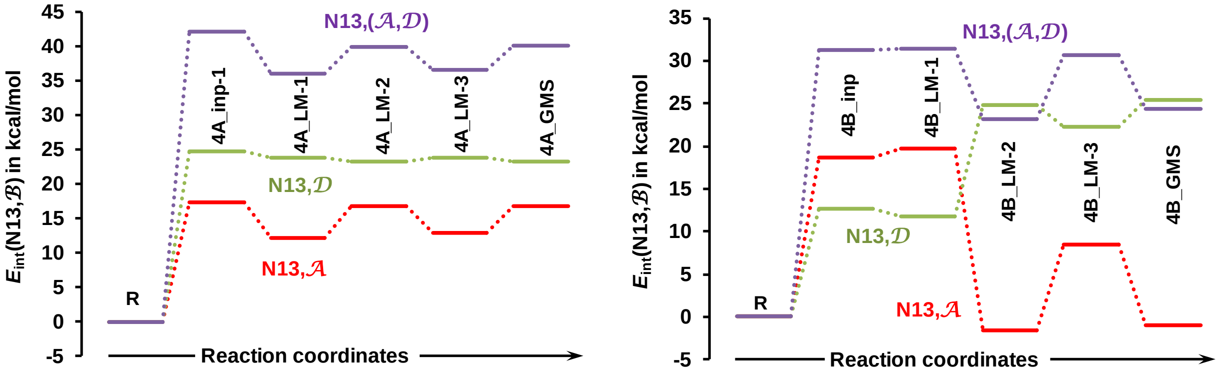

Part B

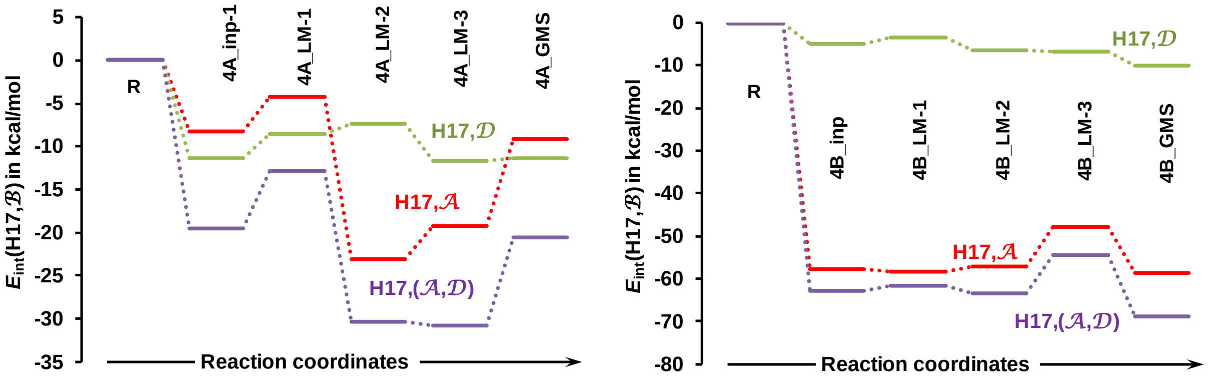

Figure S10. Interaction energies between fragments $\mathcal{A}$ (C18,O19) of acetone, $\mathcal{D}$ (S28,O37) of DMSO and the combined ($\mathcal{A}$,$\mathcal{D}$) fragments of acetone and DMSO with atoms N13 (Part A) and H17 (Part B) of proline (**1**).

Table S12. Inter-molecular interactions (in kcal/mol) between the indicated atoms of proline, either **1a** or **1b** in Part A and Part B, respectively, and molecular fragment $\mathcal{A}$ = {C18,O19} of **2** and $\mathcal{D}$ = {S28,O37} of **3** in the indicated 3-MCs.

Part A LEC

	4A_LM-3		4A_GMS	
**Atom**	$\mathcal{A}$	$\mathcal{D}$	$\mathcal{A}$	$\mathcal{D}$
C1	-6.1	-7.2	-4.7	-7.0
C4	-4.9	-6.7	-7.7	-6.4
H5	-5.0	-34.8	-7.3	-34.6
N13	13.0	23.8	13.8	23.3
C14	-25.0	-19.2	-33.2	-19.1
O15	15.8	11.6	26.4	11.7
O16	24.8	17.4	18.7	17.3
H17	-19.3	-11.6	-9.1	-11.4
C18	–	-21.6	–	-23.8
O19	–	21.6	–	18.5
S28	-6.9	–	-16.6	–
O37	6.9	–	11.3	–

Part B HEC

	4B_LM-3		4B_GMS	
**Atom**	$\mathcal{A}$	$\mathcal{D}$	$\mathcal{A}$	$\mathcal{D}$
C1	-4.3	-8.7	-5.0	-9.0
C4	-6.0	-8.5	-9.2	-7.1
H5	-8.0	-32.3	-6.2	-33.9
N13	8.5	22.2	-260.8	25.4
C14	-34.8	-29.1	-39.9	-19.6
O15	20.4	20.7	22.8	7.7
O16	33.2	13.4	34.3	17.1
H17	-47.7	-6.8	-58.6	-10.1
C18	–	-6.4	–	-16.5
O19	–	6.0	–	17.9
S28	-21.9		-12.4	–
O37	21.5		13.9	–

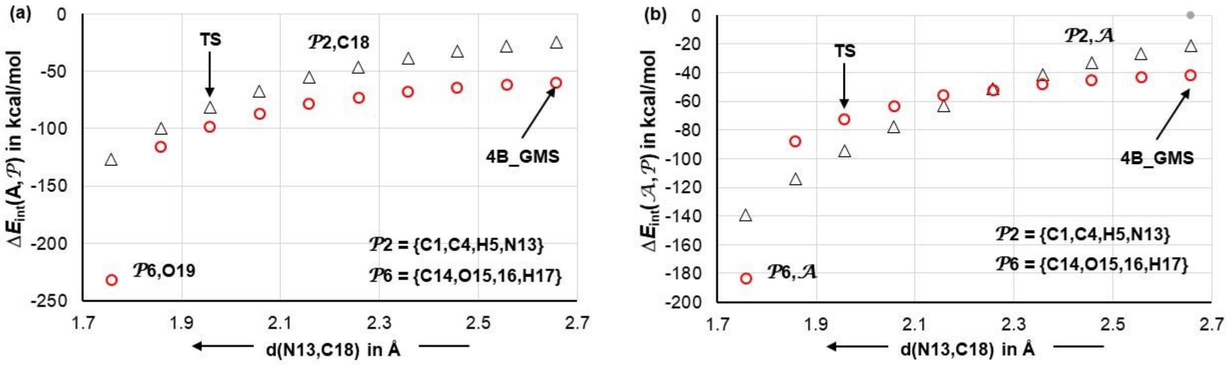

Figure S11. Trends in the interaction energy between indicated (i) atoms and a molecular fragment $\mathcal{P}$ (part a) and (ii) molecular fragment $\mathcal{A}$ and indicated molecular fragment $\mathcal{P}$ (part b). Data was obtained on simulating a CN-bond formation by scanning d(C18,13) from the value observed in the **4B_GMS** 3-MC.

End of Part 6
